# Translocation of gut bacteria promotes tumor-associated mortality by inducing immune-activated renal damage

**DOI:** 10.1038/s44318-025-00458-5

**Published:** 2025-05-22

**Authors:** Fei Cong, Hongcun Bao, Xianfeng Wang, Yang Tang, Yuwei Bao, John S Poulton, Xiaowen Liu, Adam Chun-Nin Wong, Xiang Ji, Wu-Min Deng

**Affiliations:** 1 Department of Biochemistry and Molecular Biology, Tulane University School of Medicine, Louisiana Cancer Research Center, New Orleans, LA USA; 2 Deming Department of Medicine, Tulane University School of Medicine, New Orleans, LA USA; 3 Department of Mathematics, Tulane University School of Science & Engineering, New Orleans, LA USA; 4 UNC Kidney Center, Department of Medicine, University of North Carolina at Chapel Hill, Chapel Hill, NC USA; 5 Entomology and Nematology Department, University of Florida, Gainesville, FL USA; 6 Genetics Institute, University of Florida, Gainesville, FL USA

**Keywords:** Bacterial Translocation, *Drosophila* Tumor Model, Gut-kidney Axis, Innate Immunity, Paraneoplastic Glomerulopathy, Cancer, Immunology, Microbiology, Virology & Host Pathogen Interaction

## Abstract

Paraneoplastic syndrome represents severe and complex systemic clinical symptoms manifesting in multiple organs of cancer patients, but its cause and cellular underpinnings remain little explored. In this study, establishing a *Drosophila* model of paraneoplastic syndrome triggered by tumor transplantation, we found that the innate immune response, initiated by translocated commensal bacteria from a compromised intestine, significantly contributes to reduced lifespan in tumor-bearing hosts. Our data identify the renal system as a central hub of this paraneoplastic syndrome model, wherein the pericardial nephrocytes undergo severe damage due to an elevated immune response triggered by gut dysbiosis and bacterial translocation. This innate immune response-induced nephrocyte damage is a major contributor to reduced longevity in tumor-bearing hosts, as blocking the NF-kB/Imd pathway in nephrocytes or removing gut bacteria via germ-free derivation or antibiotic treatment ameliorates nephrocyte deterioration and extends the lifespan of tumor-bearing flies. Consistently, treatment with a detoxifying drug also extended the lifespan of the tumor hosts. Our findings highlight a critical role of the gut-kidney axis in the paraneoplastic complications observed in cancer-bearing flies, suggesting potential therapeutic targets for mitigating similar complications in cancer patients.

## Synopsis

Paraneoplastic syndrome represents a major systemic complication in cancer patients but its causes remain unclear. This study establishes a novel *Drosophila* paraneoplastic-syndrome model and uncovers a critical role for gut-kidney crosstalk in shaping systemic outcomes of tumor-bearing hosts.

- Transplantation of larval tumors into adult host flies disrupts gut barrier integrity, inducing microbial dysbiosis and bacterial translocation into the body cavity.
- Translocated commensal bacteria activate innate immune Imd/NF-κB signaling in host nephrocytes.
- Immune activation in nephrocytes results in structural and functional damage, compromising their detoxification capacity.
- Ablating gut bacteria or genetically blocking immune signaling in nephrocytes reduces renal damage and prolongs survival of tumor-bearing hosts.

## Abstract (web summary)

Gut-derived inflammation and renal dysfunction are causally linked in a paraneoplastic syndrome fly model.

## Graphical abstract

**Figure:**
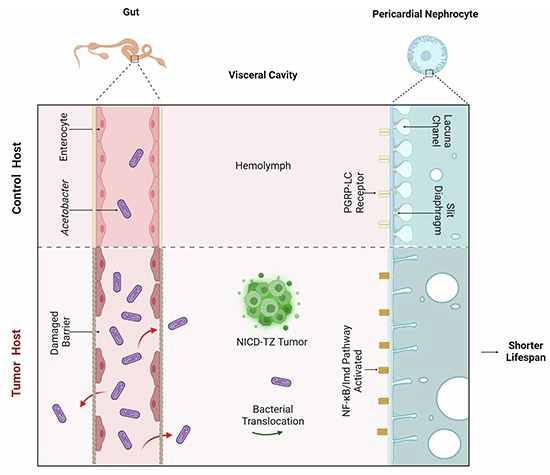

**Figure:**
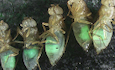

## Introduction

Paraneoplastic syndrome represents a diverse array of clinical manifestations occurring in multiple organs of cancer patients, which are not directly attributable to tumor burden or invasion (Kannoth, 2012; Sardiña González et al, 2022). The earliest documented case of paraneoplastic syndrome was reported in 1890 by French physician M. Auchè, who noted involvement of the peripheral nervous system in cancer patients (Chiu et al, 2023). Initial research primarily focused on neurological impairments caused by tumor-induced immune responses (Chiu et al, 2023; Nath and Grant, 1997). In recent years, the definition of paraneoplastic syndrome has broadened to include a wide spectrum of disorders affecting multiple organ systems, such as the cardiovascular (Kyaw et al, 2017), gastrointestinal (DiBaise, 2011), and renal systems (Lien and Lai, 2011).

There are two prevailing views regarding the causes of paraneoplastic syndromes. The first involves tumor-secreted factors, such as tumor necrosis factors (TNFs) and interleukins (ILs), which directly damage patient organs. Cancer induced cachexia, a wasting phenotype characterized by muscle loss and weight loss, is largely considered to be caused by factors released from cancer cells. Recently, researchers using *Drosophila* tumor models have shed light on the mechanisms of cachexia (Liu and Perrimon, 2022; Kwon and Song, 2015; Song et al, 2019; Ding and Song, 2021; Bilder, 2015; Hodgson et al, 2021; Newton and Hirabayashi, 2020; louise chen, 2021). The second perspective on the pathogenesis of paraneoplastic syndrome suggests that tumor-induced immune responses lead to organ damage and disrupt tissue homeostasis. For example, paraneoplastic encephalomyelitis (PEM) occurs in 75% of small cell lung cancer cases and is predominantly driven by overactivated, antigen-specific cytotoxic T cells that target neuronal structures (Soomro et al, 2020). In addition, inflammation resulting from immune activation can impact multiple organs in cancer patients, leading to conditions like vasculitis, polymyalgia rheumatica, and hypertrophic osteoarthropathy (Azar and Khasnis, 2013). Given the complexity of the mammalian immune system and the myriad triggers of immune responses, research on cancer immunity primarily focuses on the roles of specific immune cells within individual organs.

Paraneoplastic glomerulopathy is a secondary glomerular lesion syndrome driven by tumors. As the glomerulus is the basic functional unit of kidney filtration, glomerular lesions are key indicators of kidney damage. In certain cases, the diagnosis of paraneoplastic syndrome may serve as a predictor for the presence of tumors (Audard et al, 2006). An analysis of 101 individuals with chronic kidney disease revealed that 11% developed carcinoma within a decade (Lee et al, 1966). Moreover, a significant proportion of cancer patients, particularly those with lymphomas, exhibit glomerular lesions. Studies have shown that 10–27% of cancer patients display glomerular immune complex deposition in their kidneys—a hallmark of glomerular lesions detectable though immunofluorescence staining (Beaufils et al, 1985). In addition, the presence of kidney disease in cancer patients is strongly associated with poor prognosis (Launay-Vacher et al, 2016). Paraneoplastic glomerular diseases encompass a wide range of glomerular lesions, including membranous nephropathy, IgA nephropathy, and crescentic glomerulonephritis. Despite extensive research, the pathophysiological mechanisms underlying these diseases remain elusive. A leading hypothesis suggests that tumors may trigger the production of antibodies and immune complexes that are subsequently deposited in glomeruli, initiating an abnormal immune response (Kudose and Markowitz, 2019).

With the advent of advanced genetic tools and transplantation methods, a variety of tumor models have been developed in *Drosophila*. Studies on paraneoplastic syndromes in these models have focused on both tumor-secreted factors and the role of the immune response in tumor-host interactions. *Drosophila* possess only the innate immune system, which includes the immune deficiency (Imd) and Toll pathways that respond to different microbes. Compared to the response to microbial infection, the immune pathways play a paradoxical role in cancer models. For example, defensin, an anti-microbial peptide (AMP) induced by immune response can limit tumor growth in wing disc tumors of *discs large* (*dlg*) mutants (Parvy et al, 2019). In contrast, chronically activated Imd signaling in gut progenitor cells increases cell proliferation and promotes tissue hyperplasia (Petkau and Foley, 2017). Similar to certain human autoimmune diseases, an overactivated immune response in *Drosophila* also harms healthy tissues. For example, activation of the Imd and JNK pathways in the hindgut promotes extracellular matrix (ECM) damage, facilitating tumor invasion (Bangi et al, 2012). Nonetheless, the mechanisms of immune regulation and interaction across different organs remain poorly understood.

Recently, we developed a tumor model induced by the overexpression of the Notch intracellular domain (NICD), the constitutively active form of Notch, in the transition zone (TZ) of *Drosophila* larval salivary gland imaginal rings (named as NICD-TZ tumors) (Wang et al, 2021; Yang et al, 2019). These tumors exhibit many hallmarks of cancer and can be repeatedly transplanted into new host flies (Yang et al, 2019). In this study, we show that visceral growth of transplanted NICD-TZ tumors induces gut bacteria overgrowth and damages the host gut barrier, resulting in translocation of commensal bacteria. The tumor host experiences systemic and renal-specific immune responses through the Imd pathway, leading to shortened lifespan. Our findings suggest that bacterial translocation and subsequent immune activation are associated with functional decline in the fly nephrocytes, which bear molecular and functional similarity to the glomerular podocyte. Our data further demonstrates that this immune-mediated damage to the nephrocytes is a primary contributor to the reduction in lifespan in tumor hosts. Together, these findings indicate that a gut-kidney axis can contribute to paraneoplastic syndromes.

## Results

### NICD-TZ tumor transplants cause reduced lifespan and paraneoplastic defects in hosts

**Figure 1:**
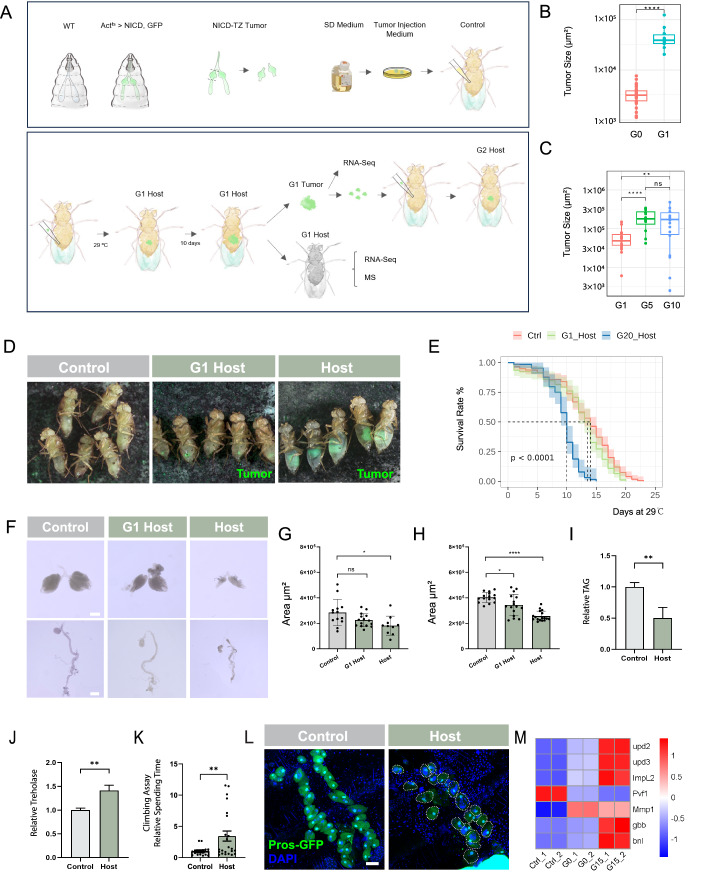
NICD-TZ tumor model and tumor host interactions. (**A**) Procedure for allografting NICD-overexpressed salivary gland transition zone tumor (Act^ts^ > NICD, GFP) into the adult fly abdomen. NICD-TZ tumors were dissected from larva and cultured in SD medium for transplantation. Flies injected with SD medium (the medium used during tumor injection) were regarded as the control flies. Flies injected with NICD-TZ tumors (G0) were regarded as G1 hosts. After resting at 25 °C for 1 day, the G1 flies were transferred to 29 °C to promote tumor tissue growth. To passage the tumor tissue, the G1 tumor was separated from the host body 10 days after transfer and was cut into small pieces, each as large as the primary tumor. These small G1 tumor pieces were then transplanted into new adult flies which become the G2 tumor hosts. Partial tumors and host bodies were used for RNA sequencing (RNA-seq) and mass-spectrometry (MS) analyses. (**B**) Size comparison of G0 tumors with G1 tumors. Box plots are defined as follows: For Primary Tumor—min: 1126, lower whisker: 1126, 25th percentile: 2426, median: 3130, mean: 3334, 75th percentile: 3808, upper whisker: 5881, max: 7606. G1 Tumor—min: 20,413, lower whisker: 20,413, 25th percentile: 33,568, median: 38,988, mean: 48,371, 75th percentile: 50,192, upper whisker: 75,127, max: 124,324.G0, *n* = 32; G1, *n* = 10. ****p = 1.4E-09. (**C**) Size comparison of G1 tumors, G5 tumors and G10 tumors. Box plots are defined as follows: G1 Tumor—min: 6047, lower whisker: 6047, 25th percentile: 37,428, median: 48,503, mean: 56,475, 75th percentile: 70,092, upper whisker: 119,087, max: 150,350. G5 Tumor—min: 42,124, lower whisker: 42,124, 25th percentile: 135,722, median: 181,204, mean: 195,632, 75th percentile: 272,787, upper whisker: 345,174, max: 345,174. G10 Tumor—min: 2505, lower whisker: 2505, 25th percentile: 70,678, median: 174,480, mean: 175,830, 75th percentile: 259,220, upper whisker: 484,617, max: 484,617. G1, *n* = 21; G5, *n* = 12; G10, *n* = 22. **p = 0.002, ****p = 3.4E-05, ns *p* = 0.6554. (**D**) Pictures of control, G1 tumor-host flies and high-passage tumor-host flies, high-passage tumor-host flies showed a reduction in white colored fat body in the tumor-host abdomens; green tissues represent the tumors. (**E**) Lifespan comparison of control flies with G1 tumor hosts and G20 hosts. Control, *n* = 121; G1-Host, *n* = 97; G20-Host, *n* = 64. ****p = 2.3E-16. Three groups were repeated. (**F**) Ovaries and guts of controls and tumor hosts 10 days after tumor injection at 29 °C. Scale bar, 2000 µm. (**G**) Quantification of the ovaries shown in (**F**). Control, *n* = 13; G1-Host, *n* = 15; G20-Host, *n* = 10. ANOVA test followed by post-hoc test. *p = 0.0157, ns *p* = 0.6894. (**H**) Quantification of the gut sizes shown in (**F**). Control, *n* = 15; G1-Host, *n* = 16; G20-Host, *n* = 17. ANOVA test followed by post-hoc test. *p = 0.0187, ****p = 1.406E-11. (**I**) Relative triglyceride levels in whole flies (tumor removed) normalized to protein amounts. Three groups were repeated. **p = 0.0095. (**J**) Relative trehalose levels in whole flies (tumor removed) normalized to extracted protein amounts. Three groups were repeated. **p = 0.0040. (**K**) Quantification of the relative time flies spend in the climbing assay. Control *n* = 19, Host *n* = 23. **p = 0.0096. (**L**) Pros-GFP marked nephrocytes in control and tumor-host flies. The white dots outline the nephrocyte shape. Scale bar, 50 µm. (**M**) Heatmap depicting representative upregulated cachectic factors within tumors of Control (wild-type tissues), G0 (larval tumors) and G15 tumors. Data is presented as mean ± SEM, Student’s t-test. Source data are available online for this figure.

To determine the longitudinal effects of tumor growth on the health of host flies, we conducted tumor transplantation studies of NICD-TZ tumors (Wang et al, 2021), which were induced in larval salivary gland imaginal ring transition zone (TZ) by over-expressing NICD with the *Act-Gal4; Gal80Ʌts* system (*Act*^*ts*^) at 29 °C. Green fluorescent protein (GFP)-labeled NICD-TZ tumors were dissected and injected into the visceral cavity of wild-type (*w*^*1118*^) female adult flies using a semi-automated device (Gong et al, 2021) (Fig. 1A). As negative controls, we also injected wild-type salivary gland imaginal rings or Schneider’s *Drosophila* (SD) medium into the visceral cavity of wild-type flies (Figs. 1A and EV1A). Since the survival assay showed no difference between these two groups (Fig. EV1B), we used SD medium-injected flies as the control group for tumor host flies. The transplanted NICD-TZ tumor grew rapidly in the visceral cavity of the host, reaching a volume ~10 times larger than the original tumor (named as generation (G) 0) after 10 days at 29 °C (Fig. 1B). To sustain tumor growth beyond the spatial constraints of the host abdomen, we dissected these transplanted tumors (G1 tumors) into smaller pieces and replanted them into new hosts to obtain G2 tumors (Fig. 1A). Using this approach, we successfully passaged the NICD-TZ tumors across numerous generations, with the longest passages persisting for about 2 years, spanning over 70 generations—well beyond the average 30-day lifespan of a fly at 29 °C. As the number of transplantation passages increased from G1 to G5, replanted tumors grew markedly larger (Figs. 1C,D and EV1C–E). While the lifespan of G1 tumor hosts showed no significant difference compared to control flies (Fig. 1E), after passage G15, the lifespan of tumor hosts was shortened to around 12 days (Figs. 1E and EV1F). To ensure consistency in our observations, all tumors and tumor hosts used in this study (unless otherwise noted) were from the G15 passage or later. In addition, we exclusively used female tumors and hosts in this study to minimize the potential influence of sex differences.

**Figure EV1:**

Low- and high- passage NICD-TZ tumors have distinct effects on the host. (**A**) Schematic diagram of the shearing of wild-type salivary-gland imaginal rings (IMR) and injection into the control host abdomen. (**B**) Comparison of the lifespan of control host flies injected with either wild-type IMR or SD medium. SD, *n* = 58; WT-IMR, *n* = 57. (**C**, **D**) The abdominal cavity of host flies carrying GFP-labeled G1 (**C**) or G43 (**D**) tumors. Scale bar, 200 µm. (**E**) Comparison of tumor sizes with different tumor passages. Box plots are defined as follows: Box plots are defined as follows: Primary Tumor—min: 1126, lower whisker: 1126, 25th percentile: 2426, median: 3130, mean: 3334, 75th percentile: 3808, upper whisker: 5881, max: 7606. No-inject—min: 0, lower whisker: 0, 25th percentile: 0, median: 0, mean: 0, 75th percentile: 0, upper whisker: 0, max: 0. G1 Tumor—min: 6047, lower whisker: 6047, 25th percentile: 37,428, median: 48,503, mean: 56,475, 75th percentile: 70,092, upper whisker: 119,087, max: 150,350. Cut Tumor—min: 841, lower whisker: 841, 25th percentile: 2844, median: 3691, mean: 3901, 75th percentile: 4640, upper whisker: 7335, max: 8026. G5 Tumor—min: 42,124, lower whisker: 42,124, 25th percentile: 135,722, median: 181,204, mean: 195,632, 75th percentile: 272,787, upper whisker: 345,174, max: 345,174. G6 Tumor—min: 1010, lower whisker: 1010, 25th percentile: 36,995, median: 72979, mean: 72,979, 75th percentile: 108,963, upper whisker: 144,947, max: 144,947. G7 Tumor—min: 24862, lower whisker: 24,862, 25th percentile: 41,071, median: 183483, mean: 165,166, 75th percentile: 234,836, upper whisker: 372,677, max: 372,677. G8 Tumor—min: 60,425, lower whisker: 60,425, 25th percentile: 87,598, median: 198,436, mean: 221,770, 75th percentile: 328,039, upper whisker: 501,919, max: 501,919. G9 Tumor—min: 33,675, lower whisker: 33,675, 25th percentile: 74,424, median: 122,889, mean: 127,351, 75th percentile: 177,309, upper whisker: 257,638, max: 257,638. G10 Tumor—min: 2505, lower whisker: 2505, 25th percentile: 70,678, median: 174,480, mean: 175,830, 75th percentile: 259,220, upper whisker: 484,617, max: 484,617.G0, *n* = 32; Non-inject, *n* = 23; G1, *n* = 32; Cut, *n* = 36; G5, *n* = 12; G6, *n* = 2; G7, *n* = 15; G8, *n* = 10; G9, *n* = 9; G10, *n* = 22. (**F**) Comparison of the lifespan of tumor host flies with increasing tumor passages. G1, *n* = 43; G10, *n* = 55; G15, *n* = 49; G20, *n* = 55; G29, *n* = 56. (**G**) Relative triglyceride levels in control and G1 host whole flies (tumor removed) normalized to protein amounts. Three groups were repeated. ns *p* = 0.1574. (**H**) Relative trehalose levels in control and G1 host whole flies (tumor removed) normalized to extracted protein amounts. Three groups were repeated. ns *p* = 0.6143. (**I**) Tumor hosts with varying abdomen sizes. Scale bar, 500 µm. Data is presented as mean ± SEM, Student’s t test. Source data are available online for this figure.

Similar to other reported *Drosophila* tumor models (Bilder, 2015; Kwon and Song, 2015), host flies bearing high-passaged NICD-TZ tumor exhibited typical organ wasting phenotypes, including smaller and degenerative ovaries (Fig. 1F,G), shorter and thinner intestines (Fig. 1F,H), and transparent abdomens, indicating a reduction of abdominal fat body (Fig. 1D). Triacylglycerols (TAG) measurement revealed a decrease in high-passaged hosts’ whole-body triglyceride storage (Fig. 1I). Another characteristic of these tumor hosts was the presence of elevated levels of trehalose (Fig. 1J), the major circulatory sugar in insects, indicating a disruption in carbohydrate metabolism that resembles hyperglycemia in cancer patients (Liu et al, 2016; Storey and Von Ah, 2015; Storey et al, 2017). By contrast, low-passage G1 host flies did not show the same intensity of changes observed in high-passage tumor hosts (Figs. 1F–H and EV1G,H). To compare transcriptomic differences between low- and high-passage tumors, we analyzed 16,355 genes and identified 1905 upregulated and 1140 downregulated genes in late-passage tumors (fold change >2). KEGG analysis of the upregulated genes revealed enrichment in pathways including spliceosome, oxidative phosphorylation, peroxisome activity, cytochrome P450, proteasome, and thiamine metabolism—processes linked to tumor progression, ROS production, and metabolic adaptation, which may contribute to host tissue damage and tumor aggressiveness (Appendix Fig. S1). Furthermore, the tumor host flies showed decreased climbing ability, suggestive of muscle degeneration (Song et al, 2019) (Fig. 1K). Interestingly, we observed that pericardial nephrocytes, the renal cells located on both sides of the heart tube (Weavers et al, 2009; Han, 2022; Koehler and Huber, 2023), were enlarged and reduced in number (Fig. 1L), suggesting a compromised renal system in these tumor hosts.

To further compare tumors with higher passage numbers (≥G15) to the control or non-passaged tumors (G0 tumor), we analyzed their gene expression profiles and found that the high-passage NICD-TZ tumors displayed significantly elevated levels of genes encoding secreted factors that could lead to organ wasting phenotypes. These include *Ecdysone-inducible gene L2* (*Impl2*), *PDGF- and VEGF-related factor 1*(*Pvf1*), *unpaired 3* (*upd3*), and *Matrix metalloproteinase 1* (*Mmp1*) (Fig. 1M), which are similar to previous reports (Kwon and Song, 2015; Song et al, 2019; Bilder, 2015; louise chen, 2021). In addition, we found that high-passage tumor hosts exhibited various degrees of abdominal bloating, a typical characteristic of cachexia (Song et al, 2019; Lee and Kwon, 2021; Kim and Bilder, 2021; Santabárbara-Ruiz and Léopold, 2021), and had increased body fluid in the abdomen compared to control flies (Figs. 1D and EV1I). Taken together, the NICD-TZ tumor transplantation model provides an ideal system for studying tumor-host interactions.

### Innate immune response in tumor hosts and the presence of bacteria in host visceral cavities

**Figure 2:**
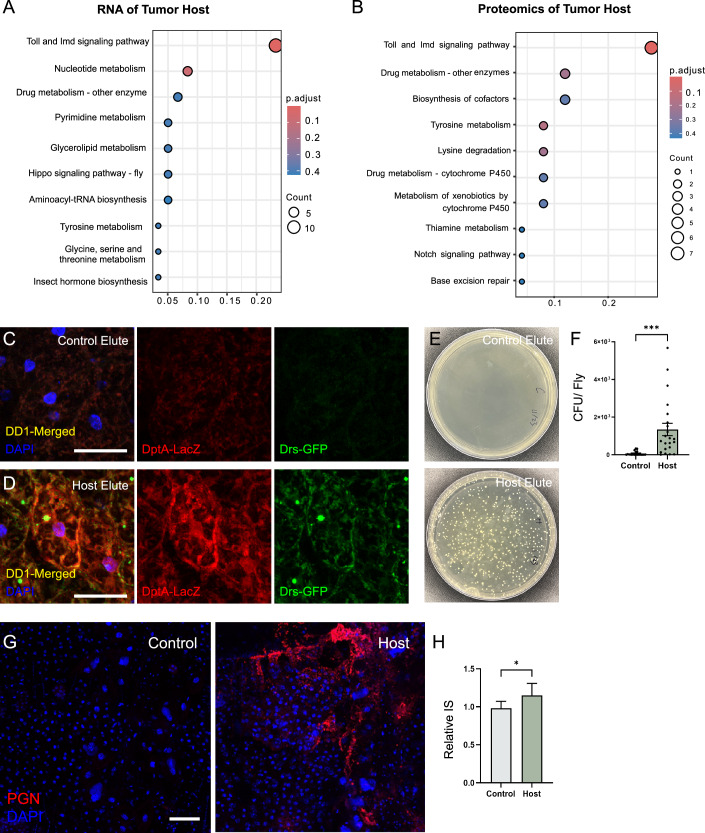
Immune response is activated in tumor hosts. (**A**, **B**) KEGG pathway enrichment analysis of tumor-host transcriptomic and proteomic data. The top 10 significant pathways are shown for differentially expressed genes between control and host. The gene ratio represents the fraction of differentially expressed genes found within the gene set. (**C**, **D**) Immunostaining of fat body from DD1 flies injected with control or tumor-host visceral hemolymph. Scale bar, 20 µm. (**E**, **F**) Bacterial loads in control and tumor-host body fluid determined by colonization number on MRS agar plates and quantification is shown in (**F**). Control, *n* = 20; Host, *n* = 22. ***p = 0.0007. (**G**) Immunostaining of PGN antibody in visceral cavity of control and tumor-host flies. Scale bar, 25 µm. (**H**) Comparison of relative IS concentration in body fluid between control and tumor-host flies. Six groups were repeated. *p = 0.0485. Data is presented as mean ± SEM, Student’s t test. Source data are available online for this figure.

To determine the transcriptomic and proteomic changes in NICD-TZ tumor hosts, we performed RNA sequencing (RNA-seq) and mass-spectrometry (MS) analyses 10 days after transplantation. Samples were acquired after removal of the tumors, and the results were compared to samples from the control flies (SD injection only). RNA-seq analysis revealed 403 differentially expressed genes (DEGs) with >1.5-fold expression difference between tumor hosts and the control (*p* < 0.05). Subsequent KEGG pathway analysis of the 363 upregulated DEGs revealed a significant enrichment of genes involved in the “Toll and Imd signaling pathway” (Fig. 2A). In the proteomics dataset (Dataset EV1), a total of 6794 proteins were detected. Among these, 138 proteins exhibited high enrichment in the tumor hosts, with a fold change greater than 1.5 (*p* < 0.05). KEGG analysis also identified the “Toll and Imd signaling pathway” as the top pathway associated with these proteins (Fig. 2B). Furthermore, by integrating RNA-seq data with proteomics data, we identified 27 genes that were upregulated in both datasets (Fig. EV2A). Notable among these are anti-microbial peptide (AMP) genes, *AttA*, *AttC*, *AttD*, and *DptB*, known to be involved in the humoral immune response (Lemaitre et al, 1997; Lemaitre and Hoffmann, 2007) (Fig. EV2B). The expression of immune-related genes did not significantly differ between untreated flies and SD-injected control flies (Fig. EV2C). These findings suggest that tumor host flies have an elevated immune response.

**Figure EV2:**

Both RNA-seq and proteomic analyses show elevated immune response in tumor hosts. (**A**) Venn diagram illustrates the overlap between upregulated genes identified through RNA-seq and proteomic analyses in tumor hosts. (**B**) List of shared upregulated genes from both RNA-seq and proteomic analyses. (**C**) Heatmap showing Imd pathway gene expression in the flies without injection (No-inject), control hosts injected with SD medium (SD-inject), and tumor hosts (Host).

To determine whether the immune response is triggered by factors in the hemolymph of the tumor host, we extracted body fluid from both control and host flies and injected them into the visceral cavity of DD1 flies, which carry two AMP reporters: *Drosomycin*-*GFP* (*Drs-GFP*) for the Toll pathway and *Diptericin A -LacZ* (*DptA-LacZ*) for the Imd pathway (Manfruelli et al, 1999). Twenty-four hours post-injection, both immune pathway reporters in the DD1 flies injected with tumor-host body fluid were strongly expressed in the fat body, the organ involved in both humoral immune response and energy homeostasis (Hoffmann and Reichhart, 2002) (Fig. 2D). In contrast, DD1 reporter expression was significantly lower in flies injected with control body fluid (Fig. 2C).

The immune response induced by the body fluid of tumor hosts led us to hypothesize the presence of bacteria in the body cavity of the hosts. To test this, we performed abdominal incisions and rinsed the internal body cavity with SD medium. The elute was then cultured on Man–Rogosa–Sharpe (MRS) agar plates (Schönborn et al, 2021). Plates with control elute exhibited almost no bacterial colonies, whereas those cultured with tumor host elute displayed a range of bacterial colonies, varying from dozens to thousands (Fig. 2E,F). To confirm that the microbes in the host body cavity were not introduced during the tumor injection processes, we harvested tumors from hosts reared on normal food conditions and injected them into axenic flies. No bacteria growth was observed in these axenic tumor hosts (*n* = 10) 10 days after tumor transplantation and culturing in a germ-free environment, suggesting that bacteria were not introduced during the injection progress. To identify these bacterial colonies, we performed PCR analysis of the 16S rDNA region followed by Sanger sequencing and found that these bacteria belong to the *Acetobacter* and *Lactiplantibacillus* families, both of which are commonly found in the fly gut (Chandler et al, 2011; Wong et al, 2011), suggesting bacterial translocation in tumor hosts.

To visualize and locate the bacterial cells within the NICD-TZ tumor host, we stained host and control abdomens with an antibody that recognizes peptidoglycan (PGN) (Demchick and Koch, 1996), a key component of the bacterial cell wall. PGN signals were detected in the abdominal tissues of tumor hosts but not in controls with SD injection (Fig. 2G). In addition, we measured the concentration of the bacterial metabolite indoxyl sulfate (IS), a well-known uremic toxin closely related to vascular and kidney diseases (Barreto et al, 2009), in the hemolymph of flies and found that the tumor hosts contained substantially higher IS levels than the controls (Fig. 2H). Collectively, these observations suggest that the immune response in tumor hosts is likely caused by the translocation of commensal bacteria into the host hemolymph.

### Gut microbial over-load and barrier dysfunction in tumor hosts

**Figure 3:**
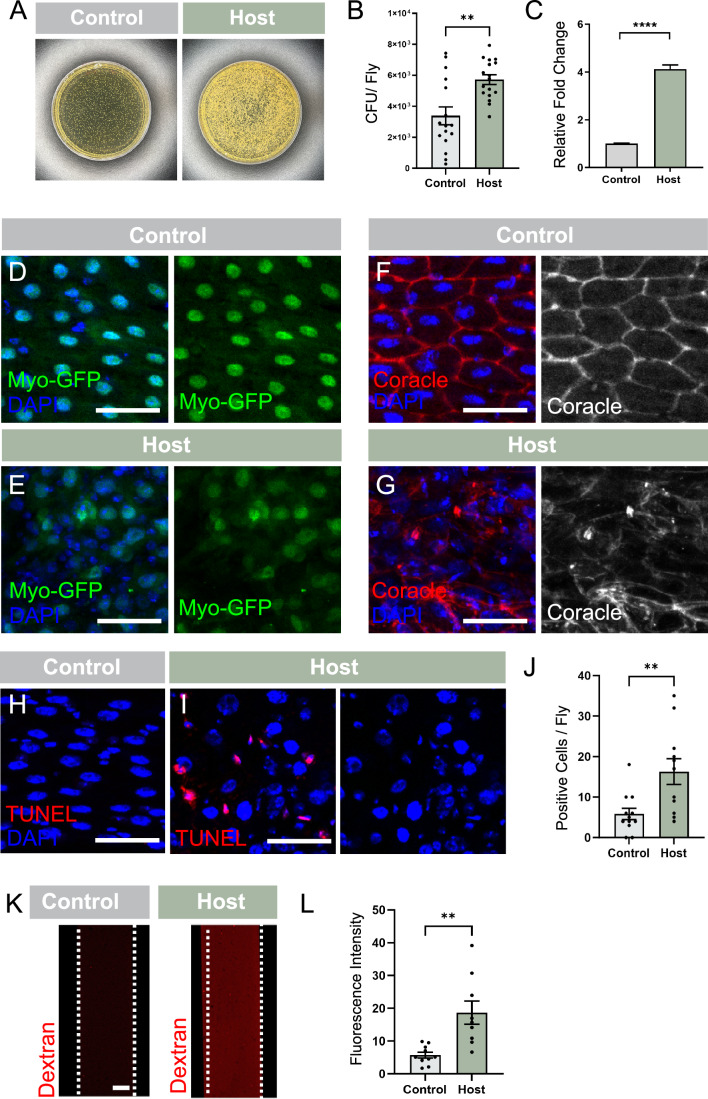
Bacteria over-load and barrier damage in host guts. (**A**, **B**) Gut bacterial load of single fly of control and tumor-host flies on MRS plates. Quantification of CFU numbers is shown in (**B**). Control, *n* = 16; Host, *n* = 17. **p = 0.001. (**C**) Relative comparison of gut bacteria numbers between control and tumor-host flies by qPCR. Three groups were repeated. ****p = 6.118E-05. (**D**, **E**) Myo1A-Gal4 > nlsGFP-labeled enterocytes (ECs) in control and tumor-host guts. Scale bar, 20 µm. (**F**, **G**) Localization of the septate junction marker, Coracle, reveals an aberrant pattern in tumor-hosts’ mid-gut. Scale bar, 20 µm. (**H**–**J**) TUNEL assay in control and tumor-host guts; quantification is shown in (**J**). Scale bar, 15 µm. Control, *n* = 12; Host, *n* = 11. **p = 0.0059. (**K**, **L**) Representative samples of hemolymph taken from control and tumor-host flies. Red fluorescence intensity reflects dextran concentration. Scale bar, 50 µm. Quantification is shown in (**L**). Control, *n* = 10; Host, *n* = 9. **p = 0.0018. Data is presented as mean ± SEM, Student’s t test. Source data are available online for this figure.

The presence of gut bacteria in the tumor host hemolymph prompted a closer examination of the host gut. First, we investigated whether the gut microbiome was altered in tumor hosts. Culturing gut bacteria from individual flies on MRS plates revealed that the host guts harbored significantly higher bacterial loads than the control (Fig. 3A,B). In addition, quantifying bacterial levels by qPCR indicated an overload of bacteria in the host gut (Fig. 3C). 16S ribosomal DNA (rDNA) sequencing of the gut microbiome showed that *Acetobacter* is the dominant population of microbiota, accounting for 95% in both control and tumor hosts (Appendix Fig. S2B). *Lactiplantibacillus*, the second largest microbiota, was slightly increased in host guts (Appendix Fig. S2B,C).

The host guts appeared remarkably shorter and thinner compared to the SD-injected controls (Fig. 1F), with the size decreasing by about 50% (Fig. 1H). The largest epithelial gut cells, enterocytes (ECs), marked by Myo-GFP, are the most abundant cells in the *Drosophila* midgut. In wild-type guts, ECs are neatly arranged, however, in tumor hosts, the arrangement of ECs was disordered, and some ECs showed decreased Myosin expression, a characteristic associated with the loss of ECs (Jiang et al, 2009) (Fig. 3D,E). The disorganization of the tumor-host gut was apparent by staining for Coracle, a component of septate junctions that delineate cell shape (Fehon et al, 1994) (Fig. 3F,G). We also performed TUNEL assay to examine cell death in the gut and found that the tumor host guts exhibited significantly higher levels of apoptotic cells (Fig. 3H–J). Furthermore, feeding flies food supplemented with fluorescent dextran showed that the fluorescent signal was significantly higher in the hemolymph of the host compared to the control (Fig. 3K,L), suggesting a leaky gut syndrome-like phenotype in tumor hosts. Together, these observations suggest that gut dysbiosis and barrier damage may have led to bacterial translocation into the hemolymph of the tumor hosts.

### Tumor-host flies have defective pericardial nephrocytes

**Figure 4:**
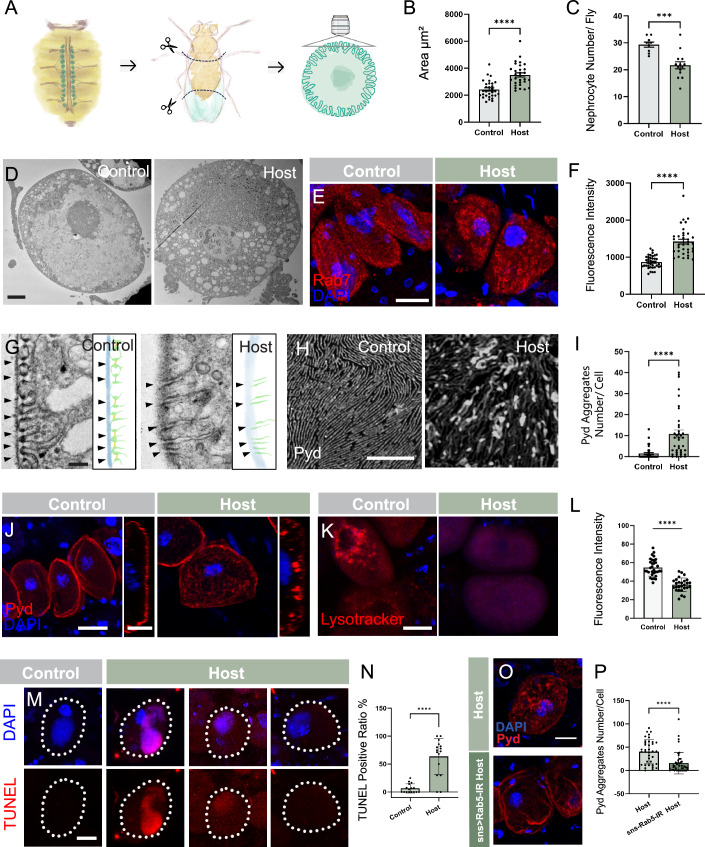
Nephrocyte damage in tumor hosts. (**A**) Schematics of pericardial nephrocyte location, dissection and imaging. (**B**) Size comparison of pericardial nephrocytes from control and tumor-host flies. Control, *n* = 30; Host, *n* = 30. ****p = 7.39E-07. (**C**) Number comparison of control and tumor-host flies pericardial nephrocytes. Control, *n* = 9; Host, *n* = 14. ***p = 0.0004. (**D**) Ultrastructural analysis of nephrocytes in control and tumor-host flies by transmission electron microscopy (TEM). Scale bar, 5 µm. (**E**) Immunostaining of Rab7 antibody in nephrocytes of control and tumor-host flies. Scale bar, 20 µm. (**F**) Quantification of Rab7 intensity in each nephrocyte of control and tumor-host flies. Control, *n* = 38; Host, *n* = 35. ****p = 7.463E-11. (**G**) TEM and schematic pictures of nephrocytes basement membrane (blue), slit diaphragm structure (yellow, arrowhead pointed) and lacunar channel (green) in control and tumor-host flies. Scale bar, 200 nm. (**H**) Pyd antibody staining pattern on the surface of nephrocytes in control and tumor-host flies with 60X lens. Scale bar, 20 nm. (**I**, **J**) Pyd location on the membrane and inside of the nephrocyte in control and tumor-host flies with 20X lens. Quantification of Pyd aggregates on the membrane of each nephrocyte is shown in (**I**). Scale bar, left 20 µm, right 10 µm. Control, *n* = 38; Host, *n* = 38. ****p = 7.006E-06. (**K**, **L**) Lysotracker staining of nephrocytes in control and tumor-host flies. Scale bar, 20 µm. Quantification of Lysotracker intensity is shown in (**L**). Control, *n* = 32; Host, *n* = 27. ****p = 1.459E-11. (**M**) TUNEL staining of nephrocytes in control and tumor host flies. The white dots outline the nephrocyte shape. Scale bar, 15 µm. (**N**) Quantification of TUNEL positive cells ratio in each fly. Control, *n* = 16; Host, *n* = 16. ****p = 1.283E-07. (**O**, **P**) Pyd staining of nephrocytes in host flies and *sns-Gal4>Rab5-RNAi* host flies. Scale bar, 20 µm. Quantification of Pyd aggregates is shown in (**P**). Control, *n* = 37; Host, *n* = 44. ****p = 2.463E-05. Data is presented as mean ± SEM, Student’s t test. Source data are available online for this figure.

Nephrocytes are a key component of the fly renal system and are molecularly and functionally similar to human podocytes. Nephrocytes play a crucial role in the filtration of hemolymph and degradation of toxic substances, processes that critically depend on endocytosis and efficient membrane vesicle transport (Weavers et al, 2009; Han, 2022). In high-passage tumor hosts, we observed significant changes in the size, number, and morphology of pericardial nephrocytes (Figs. 4A–C and 1L). To examine the subcellular structure of these nephrocytes, we performed transmission electron microscopy (TEM) analysis and found a significantly higher number of vesicles in tumor host nephrocytes compared to controls (Fig. 4D). In addition, levels of Rab7, a component of late endosomes, were increased in host nephrocytes (Fig. 4E,F). Together, these observations suggest that tumor host nephrocytes are under significant stress.

During endocytosis, when nephrocytes absorb hemolymph proteins, the endosomal cargos are subsequently transported to lysosomes for degradation, while the membrane proteins are recycled back to the cell surface (Juhász, 2016; Fu and Han, 2017; Carrasco-Rando et al, 2019). The regular function of this endocytic cycle preserves the integrity and functionality of the nephrocyte plasma membrane (Wen and Han, 2020; Ruiz-Gómez, 2021; Poulton, 2022). In nephrocytes, the majority of endocytosis occurs within repeated invaginations of the plasma membrane known as lacunar channels. These channels are sealed and stabilized by intracellular junctions known as slit diaphragms, which are remarkably similar to the specialized cell-cell junctions of human podocytes (Weavers et al, 2009). TEM examination revealed disruption of the slit diaphragms in tumor host nephrocytes (Fig. 4G, yellow). In addition, while wild-type nephrocytes have lacunar channels evenly distributed along the membrane (Fig. 4G, green), in tumor-host nephrocytes we observed a reduction in the number of lacunar channels and increase in the space between them. Another notable difference in the tumor-host nephrocytes was the indistinct basement membrane. Normally, nephrocytes are surrounded by a discrete layer of basement membrane; however, in host nephrocytes, this basement membrane became noticeably diffuse (Fig. 4G, blue).

**Figure EV3:**
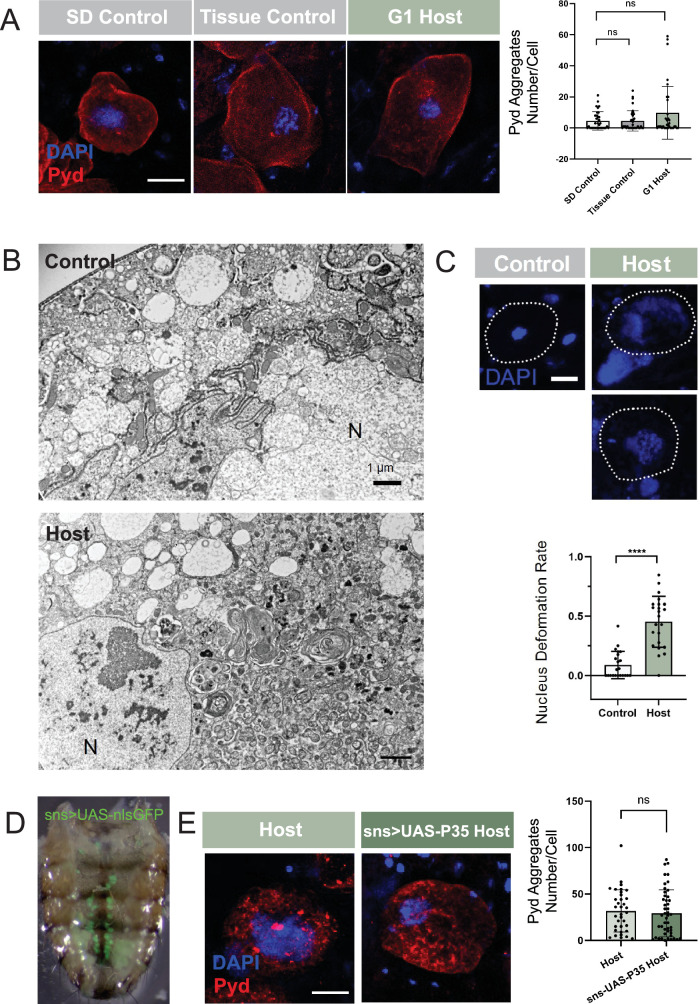
: NICD-TZ tumors cause nephrocyte damage and death in the host. (**A**) Pyd staining pattern and aggregate statistical data of SD-injected controls, Normal Tissue injected controls and G1 hosts. Scale bar, 20 µm. SD control, *n* = 34; Tissue control, *n* = 37; G1 host, *n* = 34. ns lower *p* = 0.9634. ns upper *p* = 0.0918. (**B**) TEM images of control and tumor host nephrocytes. “N” indicates nucleus. Scale bar, upper 1 µm, lower 2 µm. (**C**) Nuclear morphology in control and tumor-host nephrocytes. The white dots outline the nephrocyte shape. Scale bar, 15 µm. Quantification of defective nuclear morphology ratio in control (*n* = 22) and tumor-host flies (*n* = 23). ****p = 1.11E-08. (**D**) Expression of *sns-GAL4 > nlsGFP* in pericardial nephrocytes in adult flies. (**E**) Pyd staining pattern and aggregates statistical data of hosts and *sns-Gal4* > *UAS-P35* hosts. Scale bar, 20 µm. Host, *n* = 35; sns>P35, *n* = 47. ns *p* = 0.6423. Data is presented as mean ± SEM, Student’s t test. Source data are available online for this figure.

To further examine the structure and organization of nephrocytes in control and tumor hosts, we evaluated membrane integrity using an antibody recognizing Polychaetoid (Pyd), a component of the slit diaphragm (Carrasco-Rando et al, 2019). Located at the cytoplasmic side of slit diaphragms, Pyd staining in control nephrocytes appears in a linear pattern reminiscent of fingerprints in tangential sections (Fig. 4H). In contrast, tumor-host nephrocytes displayed an abnormal pattern of aggregations and discontinuous lines. Cross-sectional images revealed numerous Pyd-marked aggregates inside the host nephrocytes, a characteristic not observed in controls (Fig. 4J). The increased number of these anomalous Pyd aggregates indicates significant nephrocyte membrane defects in tumor hosts (Fig. 4I). The Pyd aggregates were not significantly increased in the nephrocytes of G1 hosts (Fig. EV3A).

Endocytic vesicles are essential for nephrocytes to perform their detoxification and filtering functions. The accumulation of these vesicles and aberrant Rab7 levels in tumor-host nephrocytes could indicate defects in vesicle fusion with lysosomes and problems in degrading cellular waste (Fu and Han, 2017; Wen and Han, 2020). To test this, we examined the lysosome marker Lysotracker and found that lysosomes were barely detectable in host nephrocytes (Fig. 4K,L). In addition, TEM of host nephrocytes revealed a large number of multilaminar structures located around the nucleus (Fig. EV3B). These findings suggest a decline in the waste disposal capacity of host nephrocytes, which could be detrimental to their homeostasis (Hartley and Paululat, 2018; Gould, 2021). TUNEL assay of host nephrocytes showed various levels of positive signal (Fig. 4M,N), and the ratio of nucleus deformation was also increased in these cells (Fig. EV3C), indicating ongoing cell death. Blocking nephrocyte apoptosis by over-expressing P35, which is the most widely acting anti-apoptotic protein (Hay et al, 1994), using the nephrocyte-Gal4 driver *sticks and stones (sns)-Gal4* (Zhuang et al, 2009; Zhang et al, 2013) (Fig. EV3D) did not alter the Pyd accumulation pattern (Fig. EV3E). However, when we blocked endocytosis in nephrocytes by knocking down *Rab5*, a key component of early endosomes, the mis-localization of Pyd was significantly alleviated (Fig. 4O,P). These results further suggest that nephrocyte damage is caused by a disruption in vesicle trafficking. Taking together, these data suggest that tumor hosts experience severe impairment of nephrocyte structure and function.

### Innate immune activation in nephrocytes damages them and shortens tumor host lifespan

**Figure 5:**
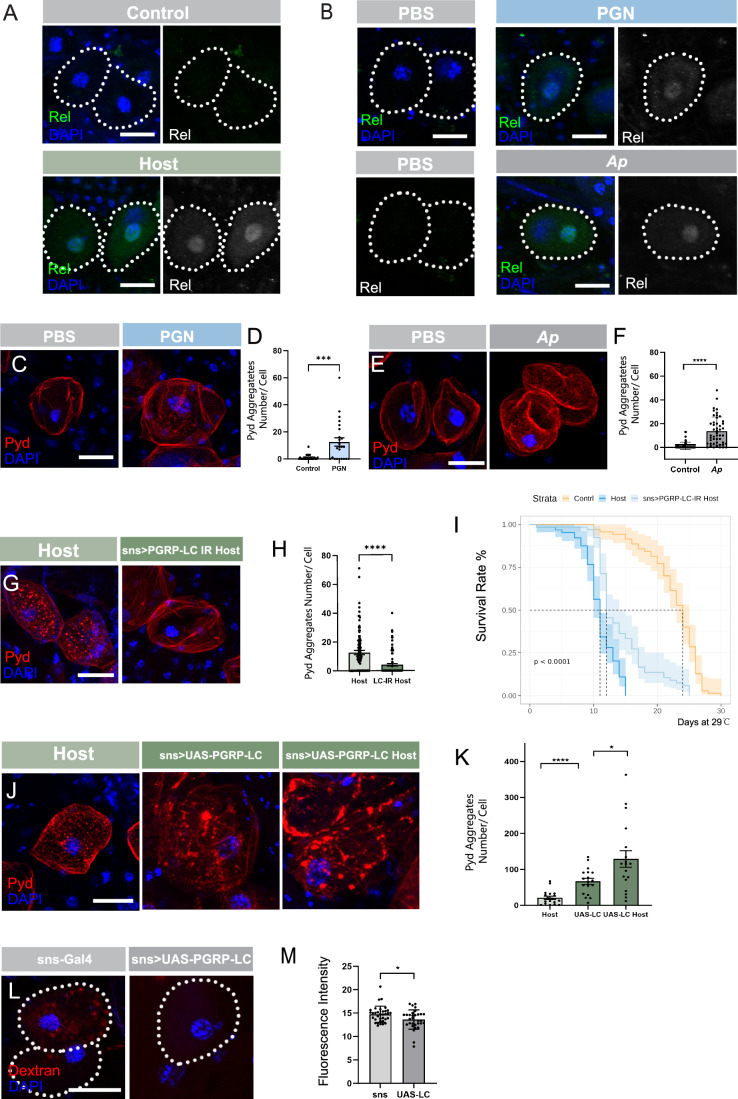
Aberrant immune response causes damage to nephrocytes. (**A**) Relish staining of nephrocytes in control and tumor host flies. The white dots outline the nephrocyte shape. Scale bar, 20 µm. Control, *n* = 42; Host, *n* = 32. (**B**) Relish staining of nephrocytes in flies injected with PBS, PGN or *Acetobacter (Ap)*. The white dots outline the nephrocyte shape. Scale bar, 20 µm. PBS *n* = 38; PGN, *n* = 36; Ap, *n* = 38. (**C**, **D**) Pyd staining of nephrocytes in flies injected with PBS or PGN. Scale bar, 20 µm. Quantification of Pyd aggregates is shown in (**D**). PBS, *n* = 24; PGN, *n* = 24. ***p = 0.0005. (**E**, **F**) Pyd staining of nephrocytes in *w1118* tumor hosts injected with PBS or *Acetobacter* (*Ap*). Scale bar, 20 µm. Quantification of Pyd aggregates is shown in (**F**). PBS, *n* = 49; *Acetobacter*, *n* = 50. ****p = 4.751E-10. (**G**, **H**) Pyd staining of nephrocytes in sns-Gal4 tumor hosts or *sns-Gal4* > *PGRP-LC-RNAi* tumor hosts. Scale bar, 20 µm. Quantification of Pyd aggregates is shown in (**H**). sns-Gal4 host, *n* = 47; *sns-Gal4* > *PGRP-LC-RNAi* host, *n* = 47. ****p = 1.283E-09. (**I**) Lifespan analysis of controls, sns-Gal4 hosts and *sns-Gal4* > *PGRP-LC-RNAi* host flies. Control, *n* = 69; sns-Gal4 host, *n* = 64; *sns-Gal4* > *PGRP-LC-RNAi* host, *n* = 66. ****p = 5.7E-08. (**J**, **K**) Pyd staining of nephrocytes in sns-Gal4 tumor hosts, *sns-Gal4* > *UAS- PGRP-LC* flies, or *sns-Gal4* > *UAS- PGRP-LC* tumor hosts. Scale bar, 20 µm. Quantification of Pyd aggregates is shown in (**K**). sns-Gal4 host, *n* = 18; *sns-Gal4* > *UAS- PGRP-LC*, *n* = 18; *sns-Gal4* > *UAS- PGRP-LC* host, *n* = 18. ANOVA test followed by post-hoc test. *p = 0.0147. ****p = 1.986E-09. (**L**, **M**) Dextran uptake assay of nephrocytes from sns-Gal4 and *sns-Gal4* > *UAS- PGRP-LC* flies. Fluorescence intensity reflects dextran concentration; quantified in (**M**). The white dots outline the nephrocyte shape. Scale bar, 20 µm. sns-Gal4, *n* = 34; *sns-Gal4* > *UAS- PGRP-LC*, *n* = 31. *p = 0.0189. Data is presented as mean ± SEM, Student’s t test. Source data are available online for this figure.

**Figure EV4:**
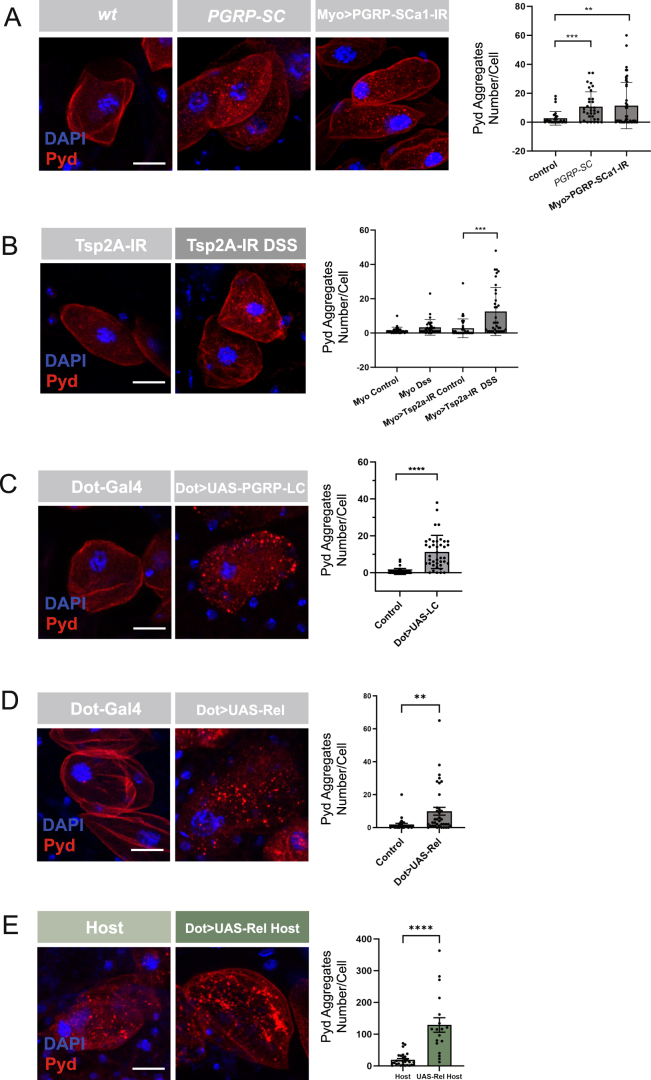
The nephrocyte immune response leads to mis-localization of Pyd on the cell membrane. (**A**) Pyd staining in wild-type flies, *PGRP-SC*^*Δ*^ mutations and *Myo1A-Gal4; Gal80∧ts* > *PGRP-SCa1-RNAi* nephrocytes. Quantification of Pyd aggregates is shown on the right. *wt*, *n* = 32; *PGRP-SC*^*Δ*^, *n* = 33; *Myo1A-Gal4; Gal80∧ts* > *PGRP-SCa1-RNAi*, *n* = 45. Scale bar, 20 µm. **p = 0.0036, ***p = 0.0001. (**B**) Pyd staining in *Myo1A-Gal4; Gal80∧ts> Tsp2A-RNAi* fly in normal food and in DSS added food. Quantification of Pyd aggregates is shown on the right. Myo control, *n* = 30; Myo DSS, *n* = 30; *Tsp2A-RNAi*, *n* = 38; *Tsp2A-RNAi* DSS, *n* = 35. Scale bar, 20 µm. ***p = 0.0002. (**C**) Pyd staining in *Dot-GAL4* and *Dot-GAL4* > *UAS-PGRP-LC* nephrocytes. Quantification of Pyd aggregates is shown on the right. Dot-GAL4, *n* = 40; *Dot-GAL4* > *UAS-PGRP-LC*, *n* = 40. Scale bar, 20 µm. ****p = 2.25E-10. (**D**) Pyd staining in *Dot-GAL4* and *Dot-GAL4* > *UAS-Relish* nephrocytes. Quantification of Pyd aggregates is shown on the right. Dot-GAL4, *n* = 29; *Dot-GAL4* > *UAS-Relish*, *n* = 37. Scale bar, 20 µm. **p = 0.0044. (**E**) Pyd staining in *Dot-Gal4* Host and *Dot-GAL4* > *UAS-Relish* tumor host nephrocytes. Quantification of Pyd aggregates is shown on the right. Dot-GAL4 host, *n* = 28; *Dot-GAL4* > *UAS-Relish* Host, *n* = 18. Scale bar, 20 µm. ****p = 5.96E-10. Data is presented as mean ± SEM, Student’s t test. Source data are available online for this figure.

Severe bacterial infections can trigger immune responses that cause kidney damage in mammals (Prasad and Patel, 2018; Doi et al, 2009). Observing bacterial translocation in high-passage tumor hosts led us to hypothesize that visceral bacteria might activate innate immune reactions in nephrocytes, thereby damaging them. *Acetobacter*, the major commensal bacterial genus in *Drosophila* is significantly amplified in tumor hosts (Appendix Fig. S2D) and was detected in host hemolymphs. *Acetobactor* are Gram-negative bacteria that can activate the Imd pathway in *Drosophila*. To determine if Imd signaling is activated in host nephrocytes, we examined the expression of Relish (Rel), the transcription factor of the pathway, which shows nuclear localization upon signaling activation. Indeed, Rel expression was detected in the nuclei of nephrocytes in tumor hosts, suggesting activation of Imd signaling in these cells (Shibata et al, 2013; Tusco et al, 2017; Snee et al, 2023) (Fig. 5A). This is consistent with the expression of Imd reporter *DptA-lacZ* in tumor hosts (Fig. 2C,D). Next, we examined whether a bacterial infection-induced immune response would affect nephrocyte membrane integrity by injecting *Acetobactor pasteurianus (Ap)* or peptidoglycan (PGN) from Gram-negative bacteria into the visceral cavity of wild-type flies. In both *Ap* and PGN-injected flies, Rel expression was detected in nephrocyte nuclei (Fig. 5B), and the Pyd expression pattern became discontinuous and formed aggregates (Fig. 5C–F). These changes suggest damage to nephrocytes, similar to the observations in tumor hosts (Fig. 4H,J). Finally, to confirm the connection between aberrant gut function and nephrocyte defects, we employed two approaches to induce gut changes and assess nephrocyte integrity. First, we knocked down the expression of the peptidoglycan recognition protein PGRP-SCa1 using a enterocyte specific gal4 (*Myo1A-Gal4; Gal80Ʌts)* (Jiang et al, 2009) or alternatively, we used the *PGRP-SC* mutants, both resulted in an increase in gut bacteria load, leading to dysbiosis in flies (Lemaitre, 2011; Guo and Jasper, 2014; Liang et al, 2023). We found in both cases, nephrocyte damage was observed, as assessed by Pyd staining (Fig. EV4A). Second, we established a gut leakage model by simultaneously knocking down the septate junction gene *Tsp2A* (Xu et al, 2019) and feeding flies with dextran sulfate sodium (DSS) (Howard et al, 2019). Under these conditions, nephrocyte damage was also observed (Fig. EV4B), suggesting a gut-nephrocyte axis in adult flies.

To determine whether Imd activation leads to nephrocyte damage, we inhibited Imd signaling in host nephrocytes by knocking down the Imd pathway receptor PGRP-LC using the *sns-Gal4* driver. In these nephrocytes, we observed a significant reduction in Pyd-positive aggregates, indicating an improvement in slit diaphragm organization (Fig. 5G,H). Interestingly, the tumor hosts with compromised Imd signaling in their nephrocytes survived longer than those with an intact immune response (Fig. 5I), suggesting a close association between nephrocyte immune response and the lifespan of the fly. Conversely, ectopic activation of Imd signaling in nephrocytes by overexpressing PGRP-LC (*sns* > *UAS-PGRP-LC*) resulted in damage to nephrocytes, as revealed by Pyd staining (Fig. 5J,K). This result was confirmed using a different nephrocyte driver, *Dorothy (Dot)-Gal4* (Gonçalves et al, 2018; Zhao et al, 2024; Kawasaki et al, 2019) (Fig. EV4C). In addition, a dextran uptake assay showed that endocytic ability decreased in Imd pathway-activated nephrocytes (Fig. 5L,M). When the *sns* > *UAS-PGRP-LC* flies were used as tumor hosts, an increase in Pyd-positive aggregates was detected (Fig. 5J,K). Similarly, overexpression of Rel with *Dot-Gal4 (Dot*>*UAS-Relish)* to activate Imd signaling resulted in comparable nephrocyte damage (Fig. EV4D). When *Dot*>*UAS-Relish* flies were used as tumor hosts, an increase in Pyd aggregates was observed, indicating enhanced slit diaphragm damage (Fig. EV4E). These observations suggest that the activation of Imd signaling in nephrocytes can affect their membrane integrity and disrupt their function.

### Reducing bacterial load improves nephrocyte morphology and prolongs lifespan of tumor hosts

**Figure 6:**
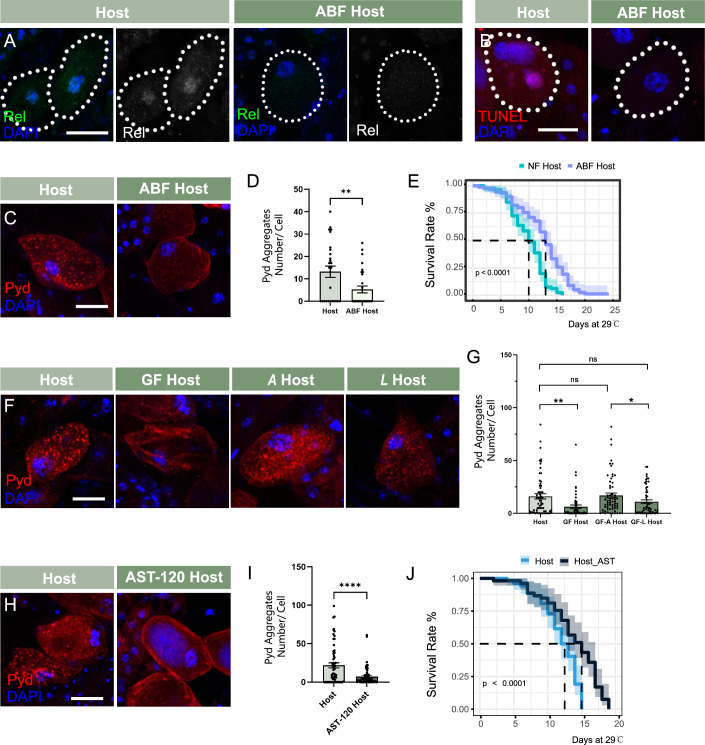
Bacteria removal alleviates nephrocyte damage and prolongs lifespan of tumor hosts. (**A**) Relish staining of nephrocytes from tumor hosts reared on normal food or antibiotic food (ABF). The white dots outline the nephrocyte shape. Control, *n* = 40; Host, *n* = 31. Scale bar, 20 µm. (**B**) TUNEL assay of nephrocytes from tumor hosts reared on normal food or antibiotic food (ABF). The white dots outline the nephrocyte shape. Control, *n* = 27; Host, *n* = 25. Scale bar, 20 µm. (**C**, **D**) Pyd staining of nephrocytes from tumor hosts reared on normal food (NF) or antibiotic food (ABF). Scale bar, 20 µm. Pyd-aggregate quantification is shown in (**D**). NF host, *n* = 27; ABF host, *n* = 29. **p = 0.0087. (**E**) Lifespan analysis of tumor hosts reared on normal food or antibiotic food. NF host, *n* = 67; ABF host, *n* = 65. ****p = 1E-08. (**F**, **G**) Pyd staining of nephrocytes from tumor hosts grown under normal conditions (Host), germ-free conditions (GF), mono-associated with *Acetobacter* host (*A* Host) or *Lactobacillus* (*L* Host). Scale bar, 20 µm. Quantification of Pyd aggregates is shown in (**G**). Host, *n* = 63; GF Host, *n* = 58; *A* Host, *n* = 63; *L* Host, *n* = 60. ANOVA test followed by post-hoc test. **p = 0.001, *p = 0.044, ns lower *p* = 0.8159, ns upper *p* = 0.0890. (**H**, **I**) Pyd staining of nephrocytes from tumor hosts reared on normal food (Host) or AST-120 supplemented food (AST-120 Host). Scale bar, 20 µm. Quantification of Pyd-aggregates is shown in (**I**). Host, *n* = 60; AST-120 Host, *n* = 68. ****p = 5.090E-05. (**J**) Survival rate of tumor hosts reared on normal food (Host) or AST-120 supplemented food (AST-120 Host). More than fifty percent control flies fed with AST-120 survived longer than 20 days (not included in Figure). Host, *n* = 50; AST-120 Host, *n* = 61. ****p = 2E-05. Data is presented as mean ± SEM, Student’s t test. Source data are available online for this figure.

**Figure EV5:**
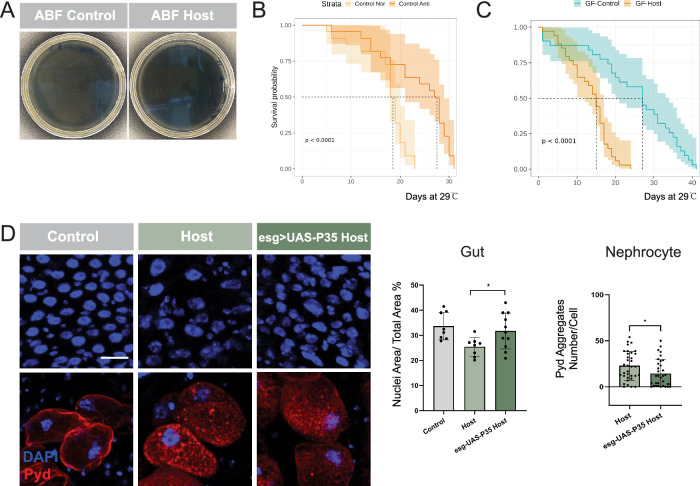
Reducing bacterial load and improving gut health benefit nephrocyte function and extend host survival time. (**A**) Intestinal bacterial load of antibiotic treated control and tumor host flies. (**B**) Lifespan of controls fed with normal food and antibiotic food. Control, *n* = 22; Control Anti, *n* = 22. ****p = 4E-05. (**C**) Lifespan of control and tumor host flies raised in germ-free conditions (GF). GF-Control, *n* = 28; GF-Host, *n* = 34. ****p = 2E-15. (**D**) DAPI staining of midgut in control, Host and *esg-Gal4; Gal80∧ts* > *UAS-P35* host flies. Quantification of nuclei densities is shown on the left. Control, *n* = 8; Host, *n* = 8; *esg* > *UAS-P35* Host, *n* = 11. Pyd staining in control, Host and *esg-Gal4; Gal80∧ts* > *UAS-P35* host nephrocytes. Quantification of Pyd aggregates is shown on the right. Host, *n* = 34; *esg* > *UAS-P35* Host, *n* = 33. Scale bar, 20 µm. Gut *p = 0.0361, Nephrocyte *p = 0.0336. Data is presented as mean ± SEM, Student’s t test. Source data are available online for this figure.

To further explore the relationship among bacterial translocation, nephrocyte immune response, and tumor-host lifespan, we administered a broad-spectrum antibiotic cocktail into the fly food (referred to as ABF) of host flies following tumor transplantation. Cultures on MRS plates from the guts of both control and tumor host flies exhibited negligible bacterial growth (Fig. EV5A), indicating effective bacterial suppression by the ABF treatment. Importantly, the ABF-treated hosts exhibited neither Rel expression nor TUNEL signal in nephrocytes (Fig. 6A,B), indicating that the mitigation of bacterial load reduced immune and apoptotic activity within these flies. In addition, the Pyd staining pattern appeared restored to normal (Fig. 6C,D). This restoration supports the hypothesis that visceral bacterial presence triggers immune responses that in turn contribute to structural damage in nephrocytes. Furthermore, the ABF-treated host flies exhibited a longer lifespan compared to those reared on normal food (Fig. 6E and EV5B). Consistently, germ-free (GF) tumor host flies, reared under axenic conditions from the egg to adult stage, showed reduced nephrocyte damage (Fig. 6F,G) and an extended lifespan (Fig. EV5C). Together, these findings indicate that reducing bacterial presence improves nephrocyte integrity and prolongs tumor host lifespan.

Next, we sought to determine whether flies mono-associated with a specific bacterial species would exhibit similar nephrocyte damage when used as tumor hosts. To this end, GF flies were cultured on medium with 10^8^ colony forming units (CFU) of either *Acetobacter pasteurianus* (*Ap*), the most common commensal bacterium in *Drosophila*, or *Lactiplantibacillus* (*Lp*), both isolated from the MRS plates with colonies grown from tumor-host abdominal fluid (Fig. 2E). These mono-associated flies and GF controls were then used as hosts of high-passage tumors. Pyd staining in the nephrocytes revealed that tumor hosts mono-associated with *Ap* showed significantly elevated levels of Pyd aggregates, whereas those associated with *Lp* exhibited less severe Pyd aggregates (Fig. 6F,G), suggesting that the Gram-negative *Acetobacter* is likely the major inducer of immune damage in tumor-host nephrocytes (Fig. 4H).

To further explore the relationship between nephrocyte function and lifespan, we fed tumor-host flies AST-120, an oral charcoal adsorbent for toxin removal that is used to treat chronic kidney disease (CKD) patients (Huang and Jinghong Zhao, 2020; Sanaka et al, 1988; Asai et al, 2019). This intervention led to noticeable improvements in the Pyd expression pattern in nephrocytes (Fig. 6H,I), indicating enhanced slit diaphragm integrity in these podocyte-like cells. Moreover, there was a significant improvement in the survival rate of the tumor-host flies (Fig. 6J). Next, we inhibited gut stem cell death by over-expressing P35 to mitigate gut damage. Statistical analysis revealed a modest reduction in Pyd aggregation (Fig. EV5D). Together, these findings suggest that alleviating the immune response by removal of bacteria or associated toxins helps prevent the deterioration of host nephrocytes and extends tumor-host lifespan.

## Discussion

Bacterial translocation, defined as the passage of gut resident bacteria to other normally sterile organs, is a common complication in cancer patients and can activate innate and adaptive immune responses (Delgado and Guddati, 2021; Yu and Schwabe, 2017). It is widely believed that dysbiosis, gut barrier defects, and immune dysfunction are necessary conditions for bacterial translocation (Dahlgren and Lennernäs, 2023). In clinical settings, chemoradiotherapy and cancer treatment drugs will aggravate the occurrence of these factors. The presence of PGN from bacteria in blood is closely associated with poor prognosis, and a significant number of cancer patients die from sepsis (Balzan et al, 2007; Potruch et al, 2022). In insects, translocation of gut bacteria into the hemolymph is linked to *B. thuringiensis* insecticidal activity of gypsy moth (Broderick et al, 2006). In the current study, we showed in a *Drosophila* tumor model that the innate immune response to translocated gut bacteria plays a critical role in paraneoplastic complications and the lifespan of tumor hosts. A crucial organ in this process is the waste-managing renal system, which is severely damaged by elevated IMD/NFκB immune signaling in tumor hosts. Blockade of the Imd pathway in nephrocytes or elimination of gut bacteria in tumor hosts ameliorated nephrocyte damage and extended the lifespan of tumor hosts. By contrast, tumor hosts mono-associated with *Acetobacter* recapitulated the pathologic immune response in nephrocytes that significantly shortened the lifespan of the tumor host. These findings suggest the crucial role of a bacteria-triggered immune response in paraneoplastic syndromes and reveal a gut-kidney interorgan communication through innate immune responses.

*Drosophila* nephrocyte are very similar to human glomerular podocytes. Both featuring specialized slit diaphragm, that filter the blood or hemolymph to remove toxic compounds (Han, 2022; Hartley and Coward, 2020). *Drosophila* nephrocytes contain orthologs for key slit diaphragm proteins, including Sns (homologous to mammalian Nephrin) and Pyd (homologous to mammalian ZO-1) (Grahammer et al, 2013). Loss of Sns or Pyd leads to structural damage of the slit diaphragm, a primary defect in many podocyte-associated diseases (Wagner et al, 2008). A complete endocytic cycle is crucial for maintaining slit diaphragm structure (Wang and Han, 2021), with Sns, Pyd and other proteins forming a complex that undergoes endocytosis at slit diaphragms. In high-passage NICD-TZ tumor hosts, the nephrocytes show aberrant slit diaphragms and accumulation of endocytic vesicles, suggesting increased uptake and incomplete membrane cycling. These damaged nephrocytes also lack lysosomes, thus the stagnation of vesicle transport in nephrocytes may contribute to the increased uremic toxin IS observed in the host hemolymph.

In cancer patients, glomerular pathologies can arise as primary conditions, or result from infections and autoimmune disorders, known as paraneoplastic glomerulopathy. Bacterial infection can also cause glomerular dysfunction through immune complex deposition in glomeruli (Satoskar et al, 2020). Previous studies indicate that NF-κB activation in podocytes contributes to glomerular injury. For instance, upregulation of NF-κB in podocytes of transgenic mice led to glomerular damage and proteinuria (Hussain et al, 2009). Conversely, inhibiting the NF-κB pathway improved kidney function after ischemia-reperfusion injury (Markó et al, 2016). These reports agree with our findings that immune response activation in nephrocytes, in particular the NF-κB/Imd pathway, perturbs nephrocyte structure and function. In addition, in the fly Malpighian tubule, the *Drosophila* renal tubule that is similar to the mammalian nephron tubule, dysbiosis also activates the NFκB/Imd pathway and disrupts metabolic homeostasis (Zugasti et al, 2020). These studies establish the fly renal system as models for kidney immune response and injury.

As the largest immune organ, the gut plays a critical role in stabilizing intestinal flora and communicating with other organs though metabolic and immune pathways (Meijers et al, 2019). The interaction between the gut and other organs is often conceptualized as, for example, the gut-liver, gut-brain, or gut-kidney axis (Yang et al, 2018). In chronic kidney disease (CKD) patients, alternations in the mucus layer and increased colon permeability have been reported, (Vaziri et al, 2012; McIntyre et al, 2011) and bacterial translocation was detected in 20% of end-stage renal disease patients though blood tests for bacteria-derived DNA fragment (Wang et al, 2012). In addition, bacterial metabolites, such as indoxyl sulfate (IS) and trimethylamine N-oxide (TMAO), are well-known uremic toxins (Lim et al, 2021). Dietary modifications or treatments that reduce IS production in a mouse model of kidney disease have been shown to mitigates kidney damage (Lobel and Garrett, 2020; Huang and Jinghong Zhao, 2020). Dysbiosis has also determined in CKD patients and in an acute kidney injury (AKI) mouse model (Vaziri et al, 2013; Yang et al, 2020). Moreover, colonizing germ-free mice with post-AKI microbiota exacerbated kidney injury, while oral antibiotics provided protection, suggesting that kidney injury maybe a consequence of microbiota alteration (Yang et al, 2020).

In our study, we found that maintaining flies in a germ-free environment or administering the toxin-absorbing drug AST-120 to tumor-bearing hosts alleviates nephrocyte damage and extends lifespan. Interestingly, similar phenotypes are observed in aging flies, which also exhibit intestinal degeneration characterized by dysbiosis, barrier disruption, and bacterial translocation (Clark et al, 2015), features that parallel those in the tumor host model. Modulating gut microbiota composition and reducing bacterial metabolites have been reported to mitigate intestinal damage and prolong lifespan in flies (Brummel et al, 2004). Together, these findings underscore the role of the kidney in preserving gut barrier integrity and regulating circulating levels of bacterially derived toxins, while also highlighting the potential nephrotoxic effects of these toxins. Although the benefits of controlling microbiota abundance are not limited to tumor-bearing flies, our results emphasize the critical importance of the gut-kidney axis in maintaining host health during tumor progression.

Nonetheless, several important questions remain. Which tumor-secreted factors disrupt gut homeostasis? Do these factors directly affect other organs, including the kidney (nephrocytes)? Beyond the kidney, which other organs targeted by innate immune responses are critical for determining the lifespan of tumor-bearing hosts? How does the NICD-TZ tumor evolve during serial transplantation? Our comparative RNA-seq analysis between low- and high-passage tumors may provide some initial clues to these questions (Appendix Fig. S1). Although definitive answers are still lacking, we believe that future multi-omics and whole-body single-cell transcriptomic studies will provide valuable insights into these mechanisms.

## Methods

**Table:** Reagents and tools table

Reagent/Resource	Reference or Source	Identifier or Catalog Number
**Experimental models**		
D. melanogaster: w[1118]	Bloomington Drosophila Stock Center	BDSC_5905
D. melanogaster: UAS-NICD	Yang et al, 2019	N/A
D. melanogaster: Act-Gal4/CyO; Gal80^ts^/TM6B	Yang et al, 2019	N/A
D. melanogaster: DD1	Bloomington Drosophila Stock Center	BDSC_55707
D. melanogaster: sns-Gal4	Bloomington Drosophila Stock Center	BDSC_92185
D. melanogaster: Dot-Gal4	Gift from Zhe Han	N/A
D. melanogaster: PGRP-LC-RNAi	Bloomington Drosophila Stock Center	BDSC_33383
D. melanogaster: UAS-PGRP-LC	Bloomington Drosophila Stock Center	BDSC_30919
D. melanogaster: UAS-Relish	Bloomington Drosophila Stock Center	BDSC_55777
D. melanogaster: Relish-RNAi	Bloomington Drosophila Stock Center	BDSC_28943
D. melanogaster: UAS-NLS::GFP	Bloomington Drosophila Stock Center	BDSC_4776
D. melanogaster: Myo1A-GAL4/CyO; UAS-GFP; Gal80ts/TM6B	Li et al, 2020	N/A
D. melanogaster: Pros-Gal4	Bloomington Drosophila Stock Center	BDSC_80572
**Antibodies**		
Mouse anti-Pyd	DSHB	PYD1 and PYD2
Mouse anti-PGN 3F6B3(10H6)	GeneTex	Cat#GTX39437
Mouse anti-Coracle	DSHB	C615.16
Mouse anti-Rab7	DSHB	Rab7
Rabbit anti-Relish	Raybiotech	Cat#130-10080
Mouse anti-β-Gal	Promega	Cat#Z3783
Alexa Fluor 488-conjugated goat anti-rabbit	Invitrogen	Cat#A11034
Alexa Fluor 555 conjugated donkey anti-mouse	Invitrogen	Cat#A32773
**Oligonucleotides and other sequence-based reagents**		
27F	AGAGTTTGATCCTGGCTCAG	For total bacteria
1492R	GGTTACCTTGTTACGACTT	For total bacteria
PASTEU-F	TCAAGTCCTCATGGCCCTTATG	For *Acetobacter*
PASTEU-R	TCGAGTTGCAGAGTGCAATCC	For *Acetobacter*
rp49F	CGTTTACTGCGGCGAGAT	
rp49R	CCGTTGGGGTTGGTGAG	
**Chemicals, Enzymes and other reagents**		
Fetal Bovine Serum (FBS)	Fisher Scientific	Cat#SH3007102
Glucose assay reagent	Megazyme	K-Gluc
Trizol	Thermo Fisher Scientific	Cat#15596018
DAPI	Thermo Fisher Scientific	Cat#D1306
Dextran, 10000 MW	Invitrogen	Cat#D22912
Dextran, 3000 MW	Invitrogen	Cat#D3329
MRS Broth	Millipore	Cat#102610656
AST-120	Kremezin, Japan	N/A
iTaq Universal SYBR Green Supermix	Bio-Rad	Cat#1725121
carbenicillin	MilliporeSigma	Cat#C1389
metronidazole	MilliporeSigma	Cat#M1547
tetracyclin	MilliporeSigma	Cat#87128
sodium hypochlorite	Clorox, Germicidal Bleach	N/A
PGN	InvivoGen	tlrl-pgnb3
**Software**		
ImageJ	National Institute of Health	https://fiji.sc/
Prism 8	GraphPad	https://www.graphpad.com/scientific-software/prism/
R (v4.0.4)	R Core Team	https://www.r-project.org/
Adobe Photoshop	Adobe	https://www.adobe.com/
Flybase		https://flybase.org/
**Other**		
glass capillary	VitroCom Technical Glass	Cat#5002
glass capillary 3.5”	Drummond Scientific	Cat#3-000-203-G/X
micropipette puller	World Precision Instruments	Cat#PUL-1000
Nanoject II	Drummond Scientific	Cat#3-000-204
glass beads	Sangon Biotech	Cat#B529319

### *Drosophila* husbandry, survival assay, and drug treatment

All flies were maintained and crossed at 25 °C on the BDSC cornmeal food with 12-h light/dark cycle, unless otherwise indicated.

The NICD-TZ tumor model was described previously (Wang et al, 2021; Yang et al, 2019). Briefly, *Act-Gal4/CyO; Gal80Ʌts/TM6B* flies were crossed with *UAS-NICD* at 25 °C. After one day egg laying, the offsprings were cultured at 18 °C for 7 days, then shifted to 29 °C for tumor induction. Only female NICD-TZ tumors were dissected for allografting at 20 days after hatching.

For antibiotic food (ABF) treatment, we followed previously described procedures (Chen and Song, 2022): Standard medium was supplemented with 150 mg/mL carbenicillin, 150 mg/mL metronidazole, and 75 mg/mL tetracyclin. Tumor-host flies were transferred to ABF one day after tumor transplantation.

For AST-120 drug treatment, the standard medium was supplemented with 5% AST-120. Before adding, AST-120 was grounded into fine powder. Tumor-hosts flies were transferred to AST-120 food one day after injection.

For survival rate assay, host flies were cultured at 29 °C and transferred to fresh food (including ABF and AST-120 food) every 2 days and the dead fly number was assessed every day. All experiments were repeated at least 3 times.

### Fly injection and tumor transplantation

Tissue transplantation has a long history in *Drosophila* (Ursprung, 1967). The detailed procedures of semi-automatic tumor transplantation were described previously (Gong et al, 2021). Briefly, 2- or 3-day-old flies were collected for allografting. After disinfecting all appliances, NICD-TZ tumors were dissected from larvae and washed in SD medium. Culturing in new SD medium, the tumors were injected into host fly abdomens by nano-injector. Tumor host controls were injected with 59.8 nL SD medium that cultured the tumors. After injection, the host flies were reared at 25 °C for one day and then incubated at 29 °C to drive tumor growth which relies on inactivation of Gal80 expression. 10 days after incubating at 29 °C, the tumor host flies were sacrificed, and tumors were harvested for the next round injection. All tumor host flies reared at 29 °C were transferred to fresh food (including ABF and AST-120 food) every 2 days.

The method of body fluid, PGN and bacteria injection were almost similar to tumor injection. 59.8 nL PGN of 1 mg/ml in PBS, 59.8 nL body fluid, or 59.8 nL bacteria of 5OD in PBS was used for single fly.

### Body fluid collection

To collect body fluid for DD1 fly injection or for bacterial culture, we first rinsed the flies with 70% ethanol several times and washed them with sterile water, then removed excess water. After disinfecting all appliances, we placed the rinsed fly on a glass slide and opened the abdomen with forceps. We then used 50 µl SD medium to irrigate the body cavity thoroughly and collected all the medium to Eppendorf tubes. The injections and bacterial culture were performed immediately after collection.

### Generation of germ-free and mono-associated flies

Axenic flies were generated by adapting the protocol published by Koyle (Wong and Chaston, 2016; Royet and Leulier, 2011). Briefly, collections of 12-h embryos were dechorionated for 1 min in 8.25% sodium hypochlorite, then washed twice in 70% ethanol and subsequently with sterile distilled water. The embryos were transferred into autoclaved food vials using a sterile brush. The axenic status of the embryos was confirmed by performing 16S qPCR on homogenates of the adult flies and by plating the homogenates on LB agar plates.

The Mono-association of germ-free (GF) flies with bacteria was conducted following the protocol outlined by Storelli (Storelli et al, 2011). Briefly, a bacterial culture (150 μl, OD600 = 1) was directly applied to the germ-free embryos and autoclaved fly food. The emerging larvae were allowed to develop on the contaminated media to the adult stage, after which the adults were transferred to new autoclaved fly food. The descendants of these adult flies are regarded as mono-associated flies.

### Immunostaining and imaging

For confocal microscope imaging, fly tissues were dissected in phosphate-buffered saline (PBS), nephrocytes were fixed in 4% formaldehyde in PBS for 20 min, and guts fixed for 1 h. After washing with PBS with 0.2% Triton X-100 (PBT), the samples were incubated in blocking buffer (PBT with 1% BSA) for 1 h then transferred to PBT with primary antibodies at 4 °C overnight with shaking. After washing in PBT three times for 15 min each, the samples incubated with second antibody at 25 °C for 1–2 h. 4 0,6-diamidino-2-phenylindole (DAPI) (1:1000) was used for nuclear staining. After 30 min of staining, samples were mounted and imaged with ZeissLSM800 confocal microscopes.

Primary antibodies were used at the following dilutions: Mouse anti-Pyd 1:50, Mouse anti-PGN 1:50, Mouse anti-Coracle 1:50, Mouse anti-Rab7 1:50, Rabbit anti-Relish 1:1000, Rabbit anti-β-Gal 1:1000. Secondary antibodies: Fluor 488-conjugated goat anti-rabbit were diluted 1:250. Cy3-conjugated donkey anti-mouse 1:500.

For gut and ovary imaging, tissues were dissected in cold PBS and mounted on glass slides, then imaged immediately.

Image analysis including fluorescent density, aggregates number quantification and area measurement were performed in ImageJ. For Pyd aggregates number quantification, we randomly selected 5 nephrocytes of each sample and more than 30 nephrocytes were analyzed for each genotype.

### TUNEL and Lysotracker assay

For TUNEL staining, we used TMR red In Situ Cell Detection Kit (Roche, Cat#12156792910) to label the apoptotic cells according to the manufacturer’s instruction.

For Lysotracker staining, the dissected nephrocytes were incubated in PBS containing 0.25 μM Lysotracker (Invitrogen, Cat#L7528) and DAPI for 5 min after dissection, then imaged by confocal microscope.

### Quantification of bacterial CFUs

Each fly gut was dissected in PBS and transferred to 100 µl SD medium. After homogenizing, 50 µl of homogenate were plated onto MRS agar plates using Glass Beads. The plates were incubated at 30 °C for 2 days, and images were taken to count colony forming unit (CFU) numbers by ImageJ.

For body fluid bacterial CFUs quantification, the irrigated medium was plated on MRS plates. The remaining steps were the same as gut bacterial CFUs quantification.

### Measurement of triglyceride, trehalose, and IS

For TAG and trehalose tests, we followed procedures described by Song (Kwon and Song, 2015) and Jie (Sun et al, 2023) by using Triglyceride Assay Kit (Abcam, Cat#ab65336) and Trehalose Assay Kit (Megazyme, Cat#K-GLU). 10-day flies were used for measurement after rearing at 29 °C. Each group contained seven hosts, and three groups were measured.

Indoxyl sulfate (IS) ELISA Kit (BlueGene, Cat#E0310039) was used for the IS assay. For each group we collected body fluid of five files, and 20 µl SD medium was used to irrigate the body cavity, as described in body fluid collection part. We then mixed all irrigated medium within a treatment together for the assay. Three groups were repeated.

### Dextran gut leakage assay

We refer to previously published methods (MacMillan and Donini, 2017). Briefly, to prepare the dextran-added food, we mixed 50 µL of a 2.5% w/v solution of fluorescently labeled dextran 3000 MW in water with 10 mg of dry yeast. After feeding 24 h, hemolymph from control and host flies were collected and loaded in rectangular glass capillaries. Then, the glass capillaries were placed on a glass slide and imaged by confocal to measure the dextran fluorescence intensity.

### Dextran uptake assay

To estimate the endocytic function of nephrocytes, we dissected cuticles with attached nephrocytes and cultured them in SD medium with 0.05 mg/ml 10,000 MW dextran for 10 min. We used PBS to wash the samples slightly and fixed them with 4% formaldehyde for 10 min. After mounting, we immediately imaged samples by confocal microscopy.

### Climbing assay

Before filming, seven flies were placed in an empty vial, and filming began after tapping them to the bottom of the vial. We recorded the time each fly spent reaching the same marked height. Three groups of each type were performed.

### qPCR

Fifteen fly guts per group were dissected and homogenized to extract DNA using standard protocols. The extracted DNA served as the template for qPCR using SYBR master mix (BIO-RAD, Cat#1725121). Bacterial 16S ribosomal DNA primers were used to amplify bacterial DNA, and Rp49 was employed to normalize gene abundance. Three technical replicates were performed for each of three biological replicates across different groups.

### 16s rDNA sequencing

Twenty fly guts were collected for the sequencing of each group. An aliquot of 30 ng of qualified genomic DNA is taken and mixed with PCR reaction reagents and fusion primers. Then library products are amplified through PCR reaction. Later, the amplified PCR products are recovered, and size selected by magnetic beads. The fragment size distribution and concentration of the library are assessed using the Agilent 2100 Bioanalyzer.

Next, the final double strand library products are denatured to generate the single strand library products. Then, the circularization reaction is set up to get single strand circularized DNA products. Any single strand linear DNA will be digested to remove. The final single strand circularized library is amplified with phi29 and rolling circle amplification (RCA) to generate the DNA nano ball (DNB) which carries multiple copies of the initial single stranded library molecule. The DNBs are loaded into the patterned nanoarray, and sequencing reads of PE250 bases length are generated with DNBSEQ-G400 platform (BGI-Shenzhen, China).

The raw sequencing data undergoes quality filtering to exclude low-quality reads, and only the remaining high-quality clean data are retained for downstream analysis. Reads are assembled into tags based on their overlap relationships. Tags are clustered into operational taxonomic units (OTUs) at a predetermined similarity threshold. These OTUs are compared against a reference database for species annotations. Subsequent analyses of sample species complexity and inter-group species differences are performed based on the OTU and species annotation results.

### Electron microscopy

For transmission electron microscopy, nephrocytes were dissected and fixed in 4% paraformaldehyde and 0.5% glutaraldehyde in 0.1 M cacodylate buffer, embedded in 2% low melting agarose, postfixed in 1% osmium tetroxide, and incubated in 1% uranyl acetate. Dehydration was performed using ethanol. After embedding in Durcupan resin, ultrathin sections were cut using a UC7 Ultramicrotome (Leica). Grids were imaged using a Leo 912 transmission electron microscope (Zeiss).

### RNA sequencing and bioinformatic analysis

Twenty flies without tumor or forty tumors of each sample were collected for the indicated generations for RNA sequencing. Total RNA was extracted using Trizol and subsequently purified using the RNA MiniPrep Kit (Zymo Research, Cat#R2072). Purified RNA samples were sequenced by Illumina HiSeq 2500 system, obtaining 40 million reads for each sample.

Raw sequencing reads were aligned to the Drosophila melanogaster genome (DBGP6.32) using STAR (v2.7.9a). Following alignment, reads were annotated using featureCounts (v2.0.0). Gene expression data were imported into the edgeR package (v3.42.4) in R (v4.4.0) for differential expression analysis. Functional enrichment analysis was conducted using the ClusterProfiler package (v4.8.3). Heatmaps of gene expression were generated using the pheatmap package (v1.0.12) with counts per million (CPM). Row scaling was applied to standardize expression values.

### Mass spectrometry analysis

Twenty flies without tumor were frozen in nitrogen and collected for each sample, and every genotype had three replicates. Proteins in the fruit fly tissue sample were extracted in 50 mM ammonium bicarbonate (pH 7.8) containing 8 M urea by sonication using 500 watts sonic dismembrator. The protein extract was reduced with 10 mM dithiothreitol at 37 °C for 30 min and then subjected to alkylation with 25 mM iodoacetamide in the dark at room temperature for 30 min. The reaction of iodoacetamide was stopped with 10 mM dithiothreitol for 10 min. The mixture was diluted 10 folds with 50 mM ammonium bicarbonate. Then 10 mM CaCl_2_ was added to the diluted mixture, which was digested overnight at 37 °C with trypsin at 1:25 (enzyme to protein). The digested sample was acidified with 10 μL of formic acid (FA), followed by shaking for 5 min and fast centrifugation. The sample mixture was desalted using a Pierce peptide desalting column, followed by drying at 30 °C with a centrifugal evaporator. The dried peptides were redissolved in water containing 0.1% FA (v/v). Peptides were separated by an UltiMate 3000 RSLC nanoLC system and then analyzed on an Orbitrap Fusion Lumos mass spectrometer (Thermo Fisher Scientific) using an overlapping window data independent acquisition mass spectrometry (DIA-MS) method.

The DIA-MS raw files were analyzed by DIA-NN (version 1.8.1) for peptide identification, protein identification and quantification. First, in-silico spectral libraries were generated from the UniProt Drosophila melanogaster proteome database (Proteome ID: UP000000803, version: 5/1/2023, entry number: 22066) using DIA-NN (v1.8.1). N-terminal methionine excision and methionine oxidation were set as variable post-translational modifications (PTMs) and carbamidomethylation of cysteines was set as the fixed PTM. Two missed cleavage sites and a maximum of three variable modifications per peptide were allowed. The peptide length was set to 6-30, and the precursor charge was set to 1-5. Second, the mass spectra in the data files were searched against the in-silico library for peptide precursor identification with the default settings. The error tolerances for the *m*/*z* values in MS1 and tandem mass spectrometry (MS/MS) spectra and the scan window size were set to automatic inference. Identified precursors were filtered with a 1% false discovery rate (FDR). In peptide precursor and protein quantification, the library-free analysis mode, cross-run normalization, the match-between-runs (MBR) method, and the top 3 precursor method were used.

### Statistical analysis

The Prism software (GraphPad) was used for statistical analyses. For qPCR experiments, we used the nonparametric Kruskal–Wallis test. We used the log-rank test Mantel-Cox for survival data analyses. Mean ± SEMs are shown. *P* value was indicated as follows: *P < 0.05, **P < 0.01, ***P < 0.001, ****P < 0.0001. ns for not significantly different.

At least three independent repeats were carried out for each experiment (unless otherwise stated in the figure legends). The total number of animals quantified, *p* values, and significance levels are indicated in the respective Figure legends.

### Graphics

Synopsis figure was created in BioRender.

## Supplementary information

Appendix

Peer Review File

Dataset EV1

Source data Fig. 1

Source data Fig. 2

Source data Fig. 3

Source data Fig. 4

Source data Fig. 5

Source data Fig. 6

EV and Appendix Figure Source Data

Expanded View Figures

## Data Availability

The datasets produced in this study are available as: PRJNA1233597. The source data of this paper are collected in the following database record: biostudies:S-SCDT-10_1038-S44318-025-00458-5.
