# Supplementary material for: Translocation of gut bacteria promotes tumor-associated mortality by inducing immune-activated renal damage
Source: EMBO J. 2025 May 22;44(13):3586–613. doi: 10.1038/s44318-025-00458-5 (PMC12217037; doi:10.1038/s44318-025-00458-5)
Supplement: Supplementary file 1 — Appendix [file 44318_2025_458_MOESM1_ESM.docx]

Contents

Appendix Figure S1 2

Appendix Figure S2 3


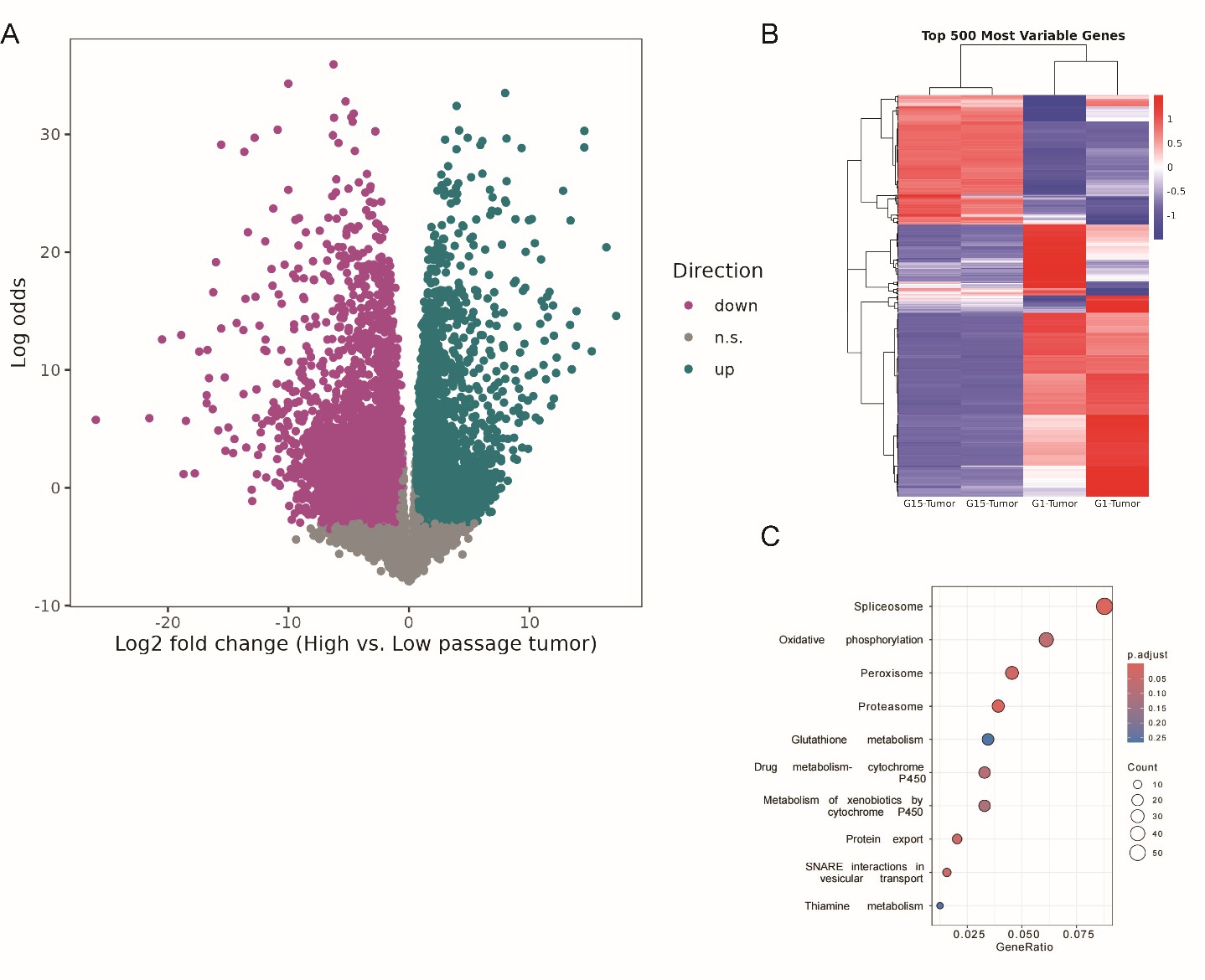


Appendix Figure S1: RNA-seq analysis of low- and high- passage NICD-TZ tumors.

1. Volcano plot shows differentially expressed genes between low-generation and high- generation tumors. Upregulated genes are shown in light blue, and downregulated genes in violet. n.s.: not significant.
2. Heatmap showing the 500 most variable genes across G1 and G15 tumors.
3. KEGG pathway analysis of genes upregulated in G15 tumors. The top 10 enriched pathways are shown.


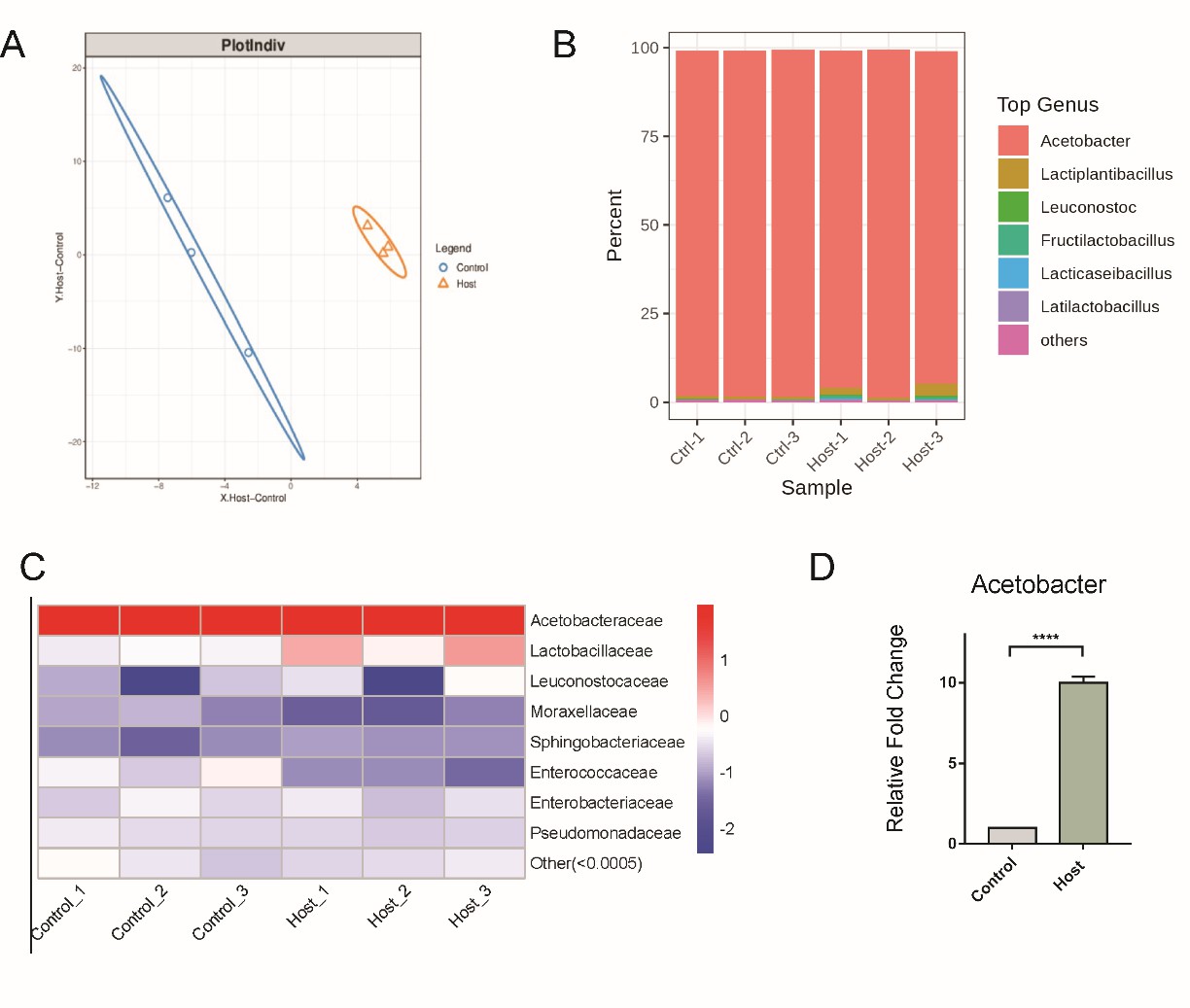


Appendix Figure S2: 16S rDNA and qPCR analysis of host gut microbiome.

1. Partial Least Squares Discriminant Analysis (PLS-DA) performed on the bacterial composition data of control and tumor host flies.
2. Composition of the top bacterial genera in SD injected control flies and tumor host flies.
3. Relative abundance heatmap of SD injected control flies and tumor-host flies.
4. Comparison of *Acetobacter* loads between control guts and host guts by qPCR.

*****p* = 2E-05. Three groups were repeated.

Data is presented as mean ± SEM, Student’s test.
