# Supplementary figures and images for: Translocation of gut bacteria promotes tumor-associated mortality by inducing immune-activated renal damage
Source: EMBO J. 2025 May 22;44(13):3586–613. doi: 10.1038/s44318-025-00458-5 (PMC12217037; doi:10.1038/s44318-025-00458-5)

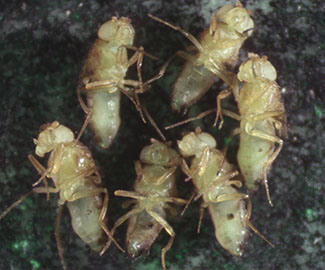

Supplement: Supplementary file 4 — Source data Fig. 1 [file 44318_2025_458_MOESM4_ESM.zip › EMBOJ-2024-119314R-SourceDataForFigure1/1D/Control.jpg]

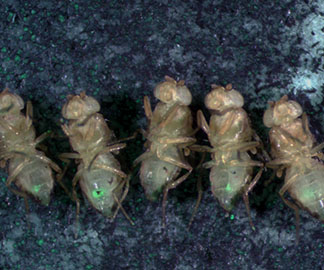

Supplement: Supplementary file 4 — Source data Fig. 1 [file 44318_2025_458_MOESM4_ESM.zip › EMBOJ-2024-119314R-SourceDataForFigure1/1D/G1 Host.jpg]

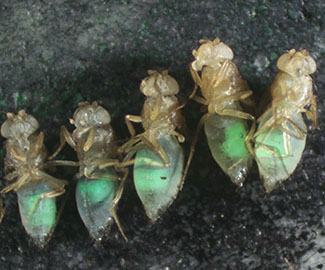

Supplement: Supplementary file 4 — Source data Fig. 1 [file 44318_2025_458_MOESM4_ESM.zip › EMBOJ-2024-119314R-SourceDataForFigure1/1D/Host.jpg]

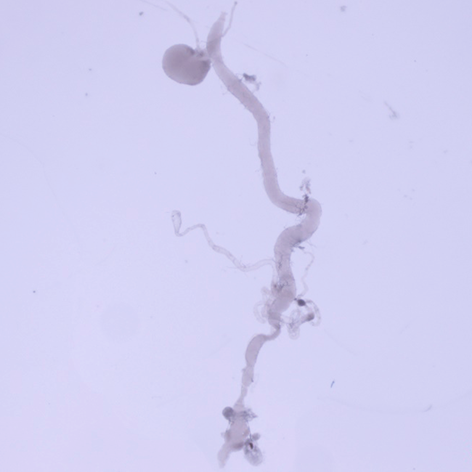

Supplement: Supplementary file 4 — Source data Fig. 1 [file 44318_2025_458_MOESM4_ESM.zip › EMBOJ-2024-119314R-SourceDataForFigure1/1F/Control Gut.tif]

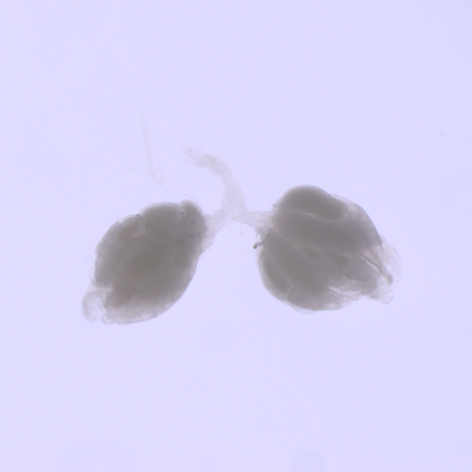

Supplement: Supplementary file 4 — Source data Fig. 1 [file 44318_2025_458_MOESM4_ESM.zip › EMBOJ-2024-119314R-SourceDataForFigure1/1F/Control Ovary.tif]

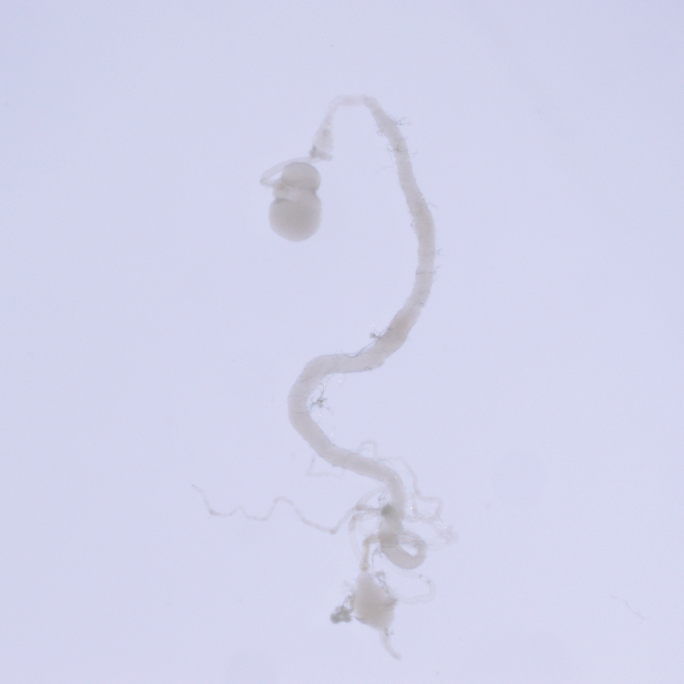

Supplement: Supplementary file 4 — Source data Fig. 1 [file 44318_2025_458_MOESM4_ESM.zip › EMBOJ-2024-119314R-SourceDataForFigure1/1F/G1 Host Gut.tif]

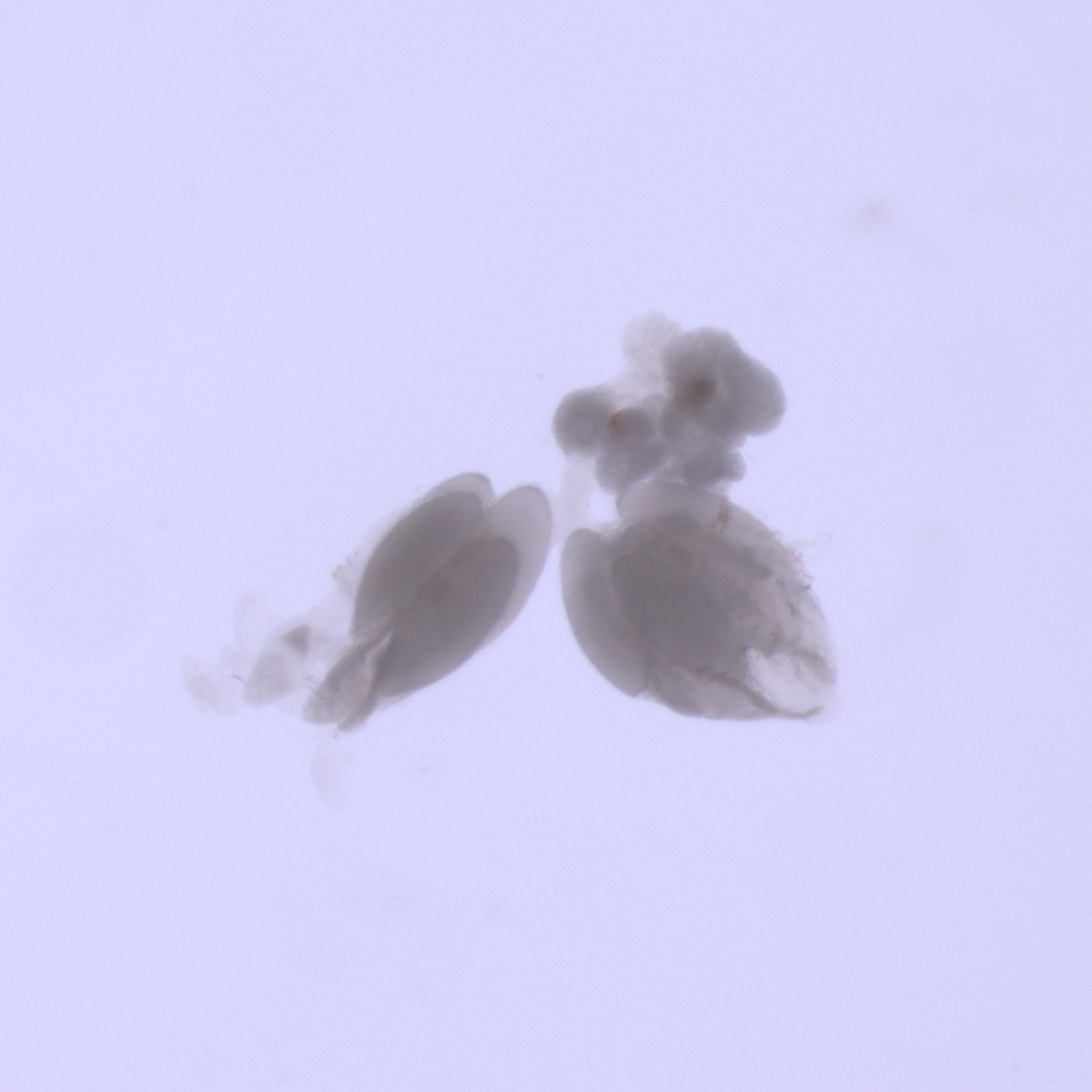

Supplement: Supplementary file 4 — Source data Fig. 1 [file 44318_2025_458_MOESM4_ESM.zip › EMBOJ-2024-119314R-SourceDataForFigure1/1F/G1 Host Ovary.tif]

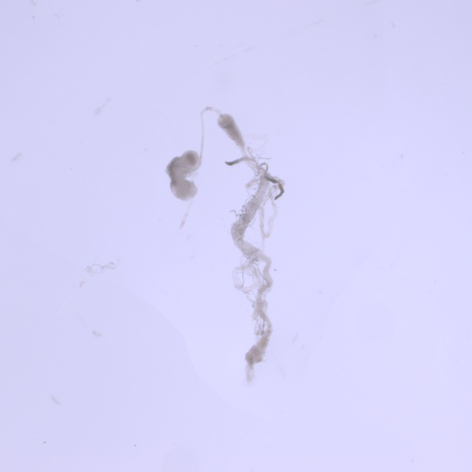

Supplement: Supplementary file 4 — Source data Fig. 1 [file 44318_2025_458_MOESM4_ESM.zip › EMBOJ-2024-119314R-SourceDataForFigure1/1F/Host Gut.tif]

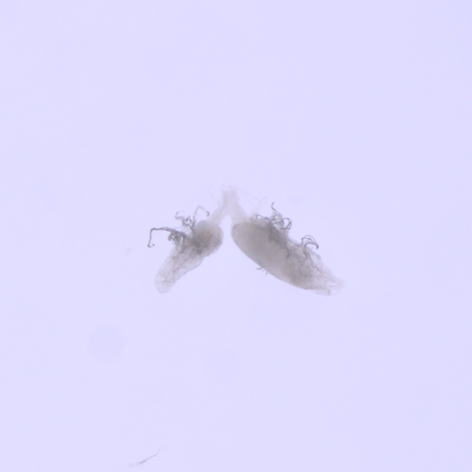

Supplement: Supplementary file 4 — Source data Fig. 1 [file 44318_2025_458_MOESM4_ESM.zip › EMBOJ-2024-119314R-SourceDataForFigure1/1F/Host Ovary.tif]

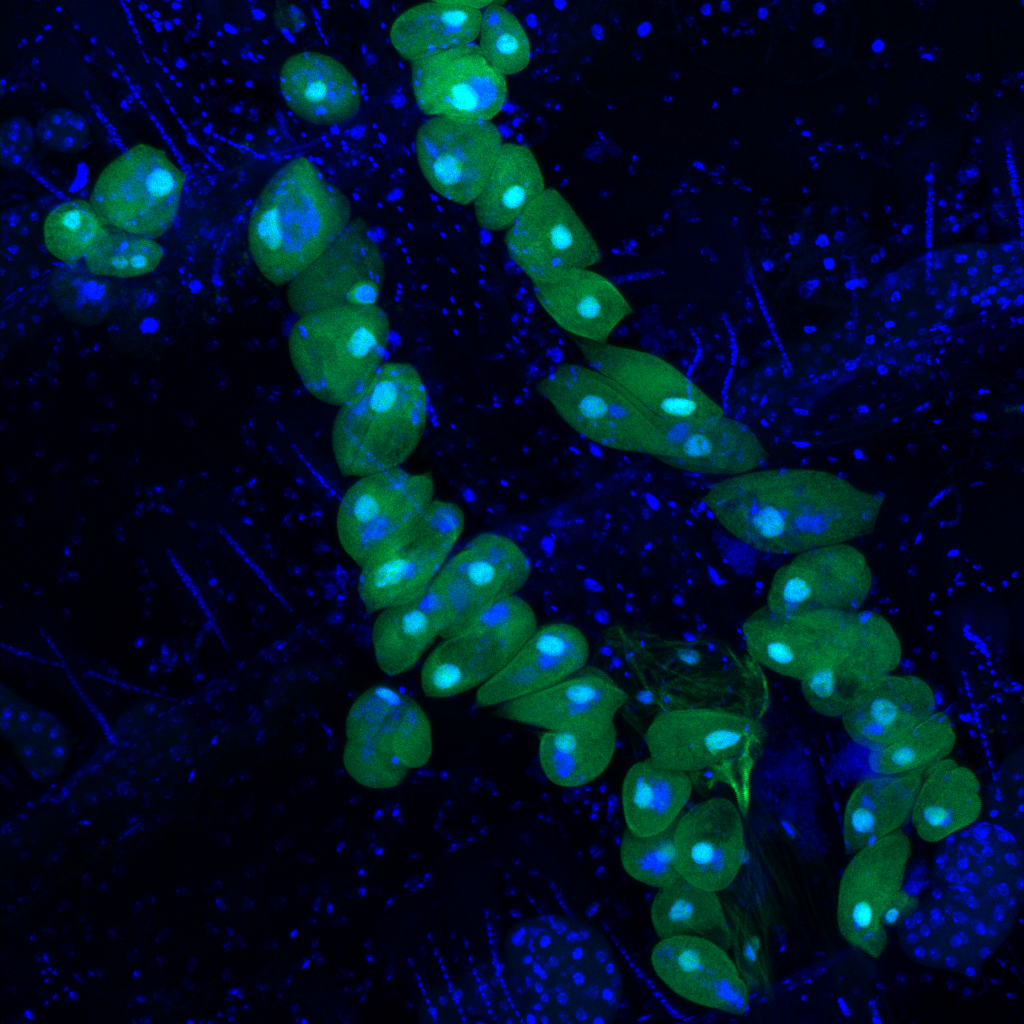

Supplement: Supplementary file 4 — Source data Fig. 1 [file 44318_2025_458_MOESM4_ESM.zip › EMBOJ-2024-119314R-SourceDataForFigure1/1L/Control.tif]

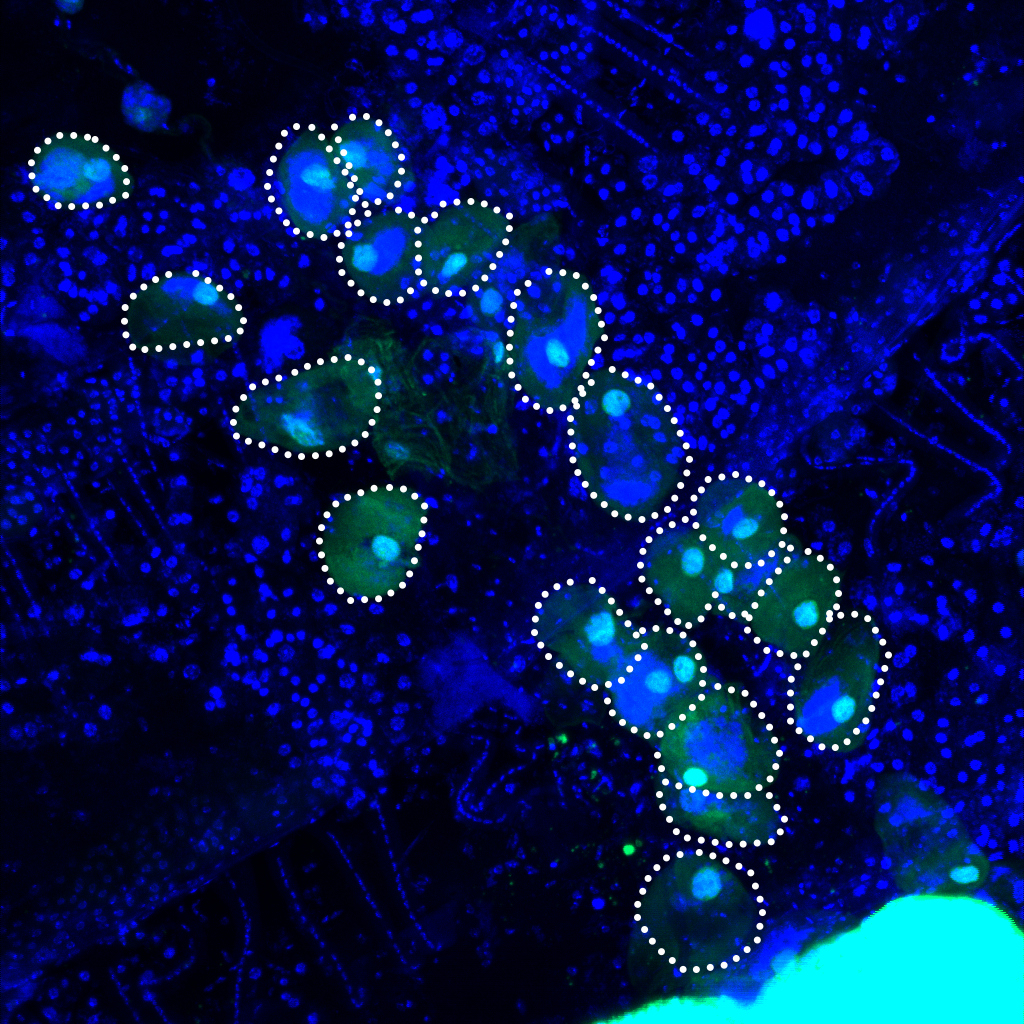

Supplement: Supplementary file 4 — Source data Fig. 1 [file 44318_2025_458_MOESM4_ESM.zip › EMBOJ-2024-119314R-SourceDataForFigure1/1L/Host.tif]

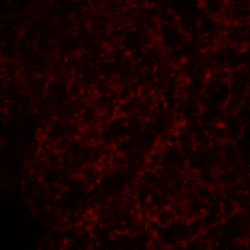

Supplement: Supplementary file 5 — Source data Fig. 2 [file 44318_2025_458_MOESM5_ESM.zip › EMBOJ-2024-119314R-SourceDataForFigure2/2C/DD1 control DptA-Lacz.tif]

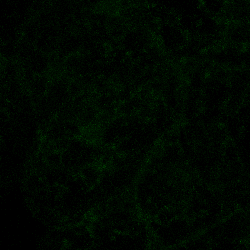

Supplement: Supplementary file 5 — Source data Fig. 2 [file 44318_2025_458_MOESM5_ESM.zip › EMBOJ-2024-119314R-SourceDataForFigure2/2C/DD1 control Drs-GFP.tif]

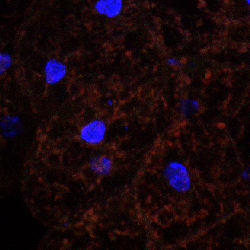

Supplement: Supplementary file 5 — Source data Fig. 2 [file 44318_2025_458_MOESM5_ESM.zip › EMBOJ-2024-119314R-SourceDataForFigure2/2C/DD1 control merged.tif]

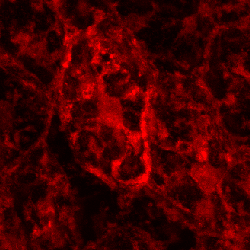

Supplement: Supplementary file 5 — Source data Fig. 2 [file 44318_2025_458_MOESM5_ESM.zip › EMBOJ-2024-119314R-SourceDataForFigure2/2D/DD1 host DptA-Lacz.tif]

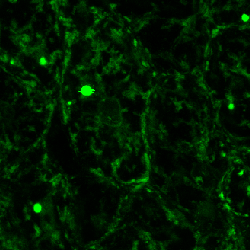

Supplement: Supplementary file 5 — Source data Fig. 2 [file 44318_2025_458_MOESM5_ESM.zip › EMBOJ-2024-119314R-SourceDataForFigure2/2D/DD1 host Drs-GFP.tif]

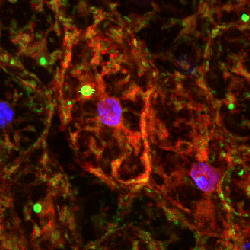

Supplement: Supplementary file 5 — Source data Fig. 2 [file 44318_2025_458_MOESM5_ESM.zip › EMBOJ-2024-119314R-SourceDataForFigure2/2D/DD1 host merged.tif]

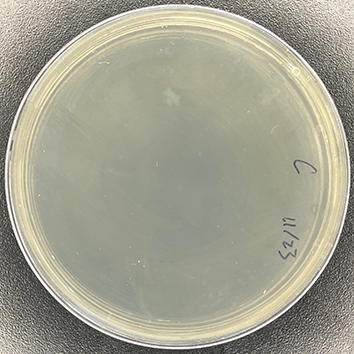

Supplement: Supplementary file 5 — Source data Fig. 2 [file 44318_2025_458_MOESM5_ESM.zip › EMBOJ-2024-119314R-SourceDataForFigure2/2E/Control.tif]

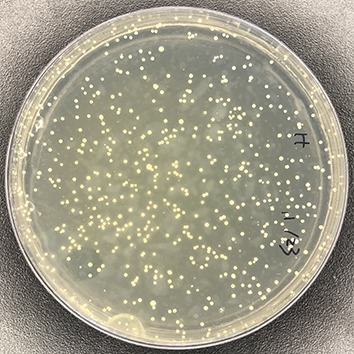

Supplement: Supplementary file 5 — Source data Fig. 2 [file 44318_2025_458_MOESM5_ESM.zip › EMBOJ-2024-119314R-SourceDataForFigure2/2E/Host.tif]

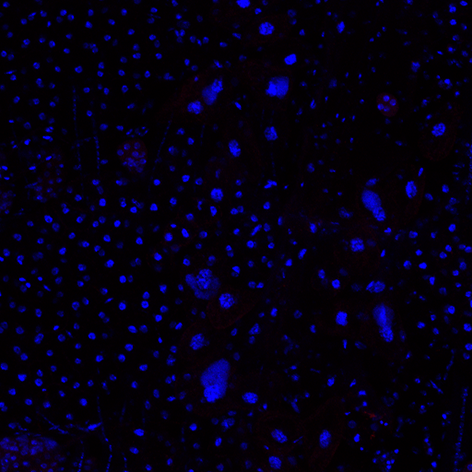

Supplement: Supplementary file 5 — Source data Fig. 2 [file 44318_2025_458_MOESM5_ESM.zip › EMBOJ-2024-119314R-SourceDataForFigure2/2G/Control PGN.tif]

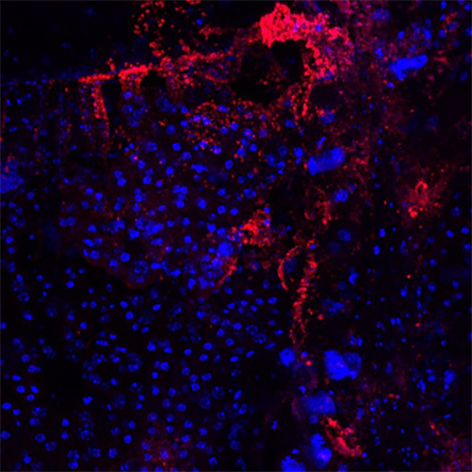

Supplement: Supplementary file 5 — Source data Fig. 2 [file 44318_2025_458_MOESM5_ESM.zip › EMBOJ-2024-119314R-SourceDataForFigure2/2G/Host PGN.tif]

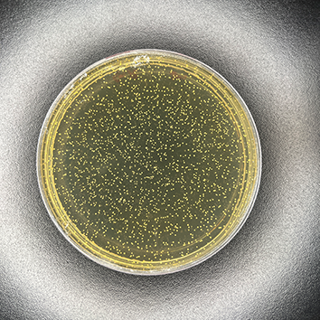

Supplement: Supplementary file 6 — Source data Fig. 3 [file 44318_2025_458_MOESM6_ESM.zip › EMBOJ-2024-119314R-SourceDataForFigure3/3A/Control.tif]

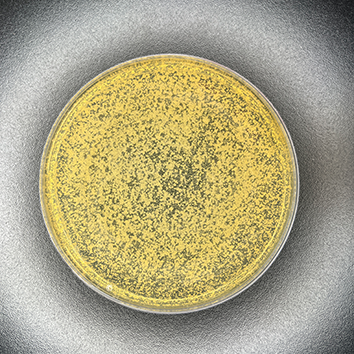

Supplement: Supplementary file 6 — Source data Fig. 3 [file 44318_2025_458_MOESM6_ESM.zip › EMBOJ-2024-119314R-SourceDataForFigure3/3A/Host.tif]

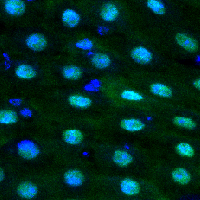

Supplement: Supplementary file 6 — Source data Fig. 3 [file 44318_2025_458_MOESM6_ESM.zip › EMBOJ-2024-119314R-SourceDataForFigure3/3D/Control Merged.tif]

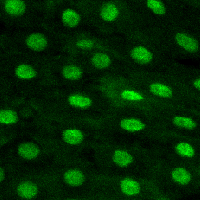

Supplement: Supplementary file 6 — Source data Fig. 3 [file 44318_2025_458_MOESM6_ESM.zip › EMBOJ-2024-119314R-SourceDataForFigure3/3D/Control Myo-GFP.tif]

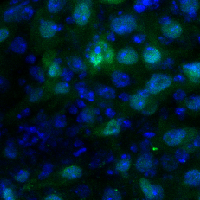

Supplement: Supplementary file 6 — Source data Fig. 3 [file 44318_2025_458_MOESM6_ESM.zip › EMBOJ-2024-119314R-SourceDataForFigure3/3E/Host Merged.tif]

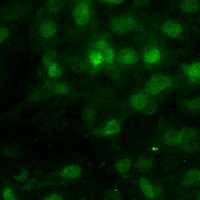

Supplement: Supplementary file 6 — Source data Fig. 3 [file 44318_2025_458_MOESM6_ESM.zip › EMBOJ-2024-119314R-SourceDataForFigure3/3E/Host Myo-GFP.tif]

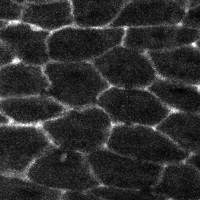

Supplement: Supplementary file 6 — Source data Fig. 3 [file 44318_2025_458_MOESM6_ESM.zip › EMBOJ-2024-119314R-SourceDataForFigure3/3F/Control Coracle.tif]

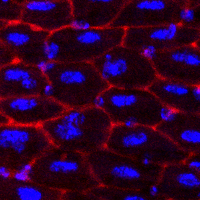

Supplement: Supplementary file 6 — Source data Fig. 3 [file 44318_2025_458_MOESM6_ESM.zip › EMBOJ-2024-119314R-SourceDataForFigure3/3F/Control Merged.tif]

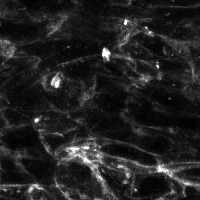

Supplement: Supplementary file 6 — Source data Fig. 3 [file 44318_2025_458_MOESM6_ESM.zip › EMBOJ-2024-119314R-SourceDataForFigure3/3G/Host Coracle.tif]

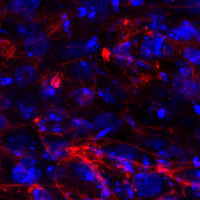

Supplement: Supplementary file 6 — Source data Fig. 3 [file 44318_2025_458_MOESM6_ESM.zip › EMBOJ-2024-119314R-SourceDataForFigure3/3G/Host Merged.tif]

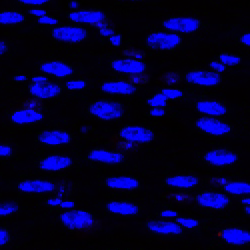

Supplement: Supplementary file 6 — Source data Fig. 3 [file 44318_2025_458_MOESM6_ESM.zip › EMBOJ-2024-119314R-SourceDataForFigure3/3H/Control Tunel.tif]

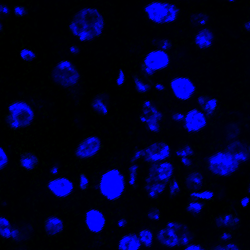

Supplement: Supplementary file 6 — Source data Fig. 3 [file 44318_2025_458_MOESM6_ESM.zip › EMBOJ-2024-119314R-SourceDataForFigure3/3I/Host DAPI.tif]

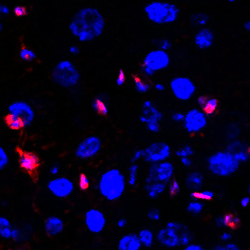

Supplement: Supplementary file 6 — Source data Fig. 3 [file 44318_2025_458_MOESM6_ESM.zip › EMBOJ-2024-119314R-SourceDataForFigure3/3I/Host Tunel Merged.tif]

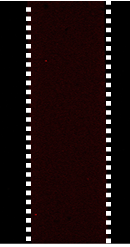

Supplement: Supplementary file 6 — Source data Fig. 3 [file 44318_2025_458_MOESM6_ESM.zip › EMBOJ-2024-119314R-SourceDataForFigure3/3K/control dextran.png]

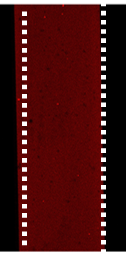

Supplement: Supplementary file 6 — Source data Fig. 3 [file 44318_2025_458_MOESM6_ESM.zip › EMBOJ-2024-119314R-SourceDataForFigure3/3K/host dextran.png]

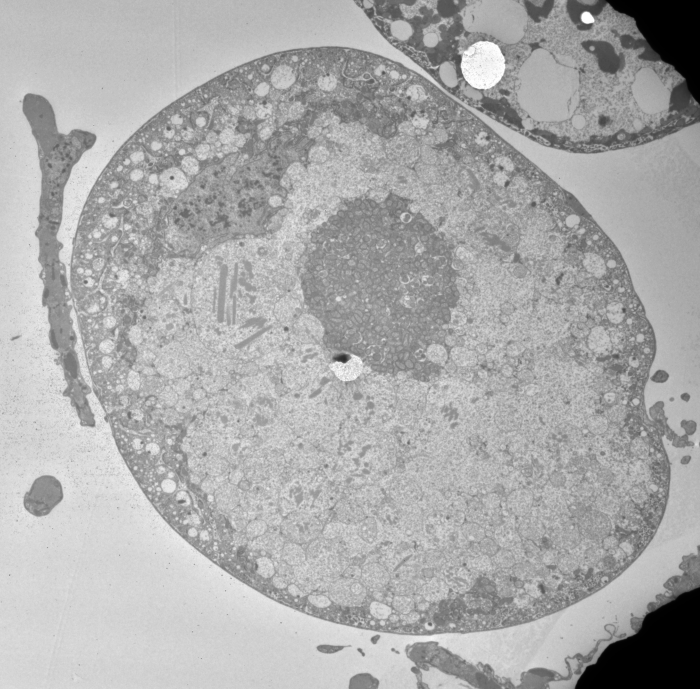

Supplement: Supplementary file 7 — Source data Fig. 4 [file 44318_2025_458_MOESM7_ESM.zip › EMBOJ-2024-119314R-SourceDataForFigure4/4D/Control.tif]

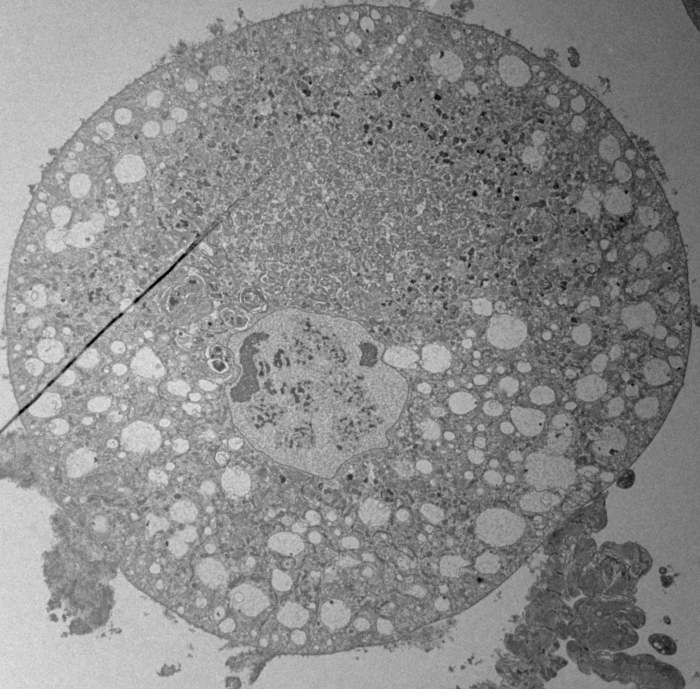

Supplement: Supplementary file 7 — Source data Fig. 4 [file 44318_2025_458_MOESM7_ESM.zip › EMBOJ-2024-119314R-SourceDataForFigure4/4D/Host.tif]

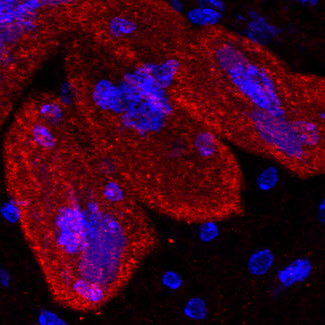

Supplement: Supplementary file 7 — Source data Fig. 4 [file 44318_2025_458_MOESM7_ESM.zip › EMBOJ-2024-119314R-SourceDataForFigure4/4E/Control.tif]

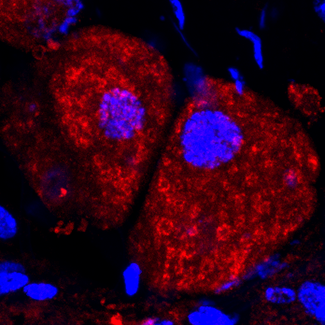

Supplement: Supplementary file 7 — Source data Fig. 4 [file 44318_2025_458_MOESM7_ESM.zip › EMBOJ-2024-119314R-SourceDataForFigure4/4E/Host.tif]

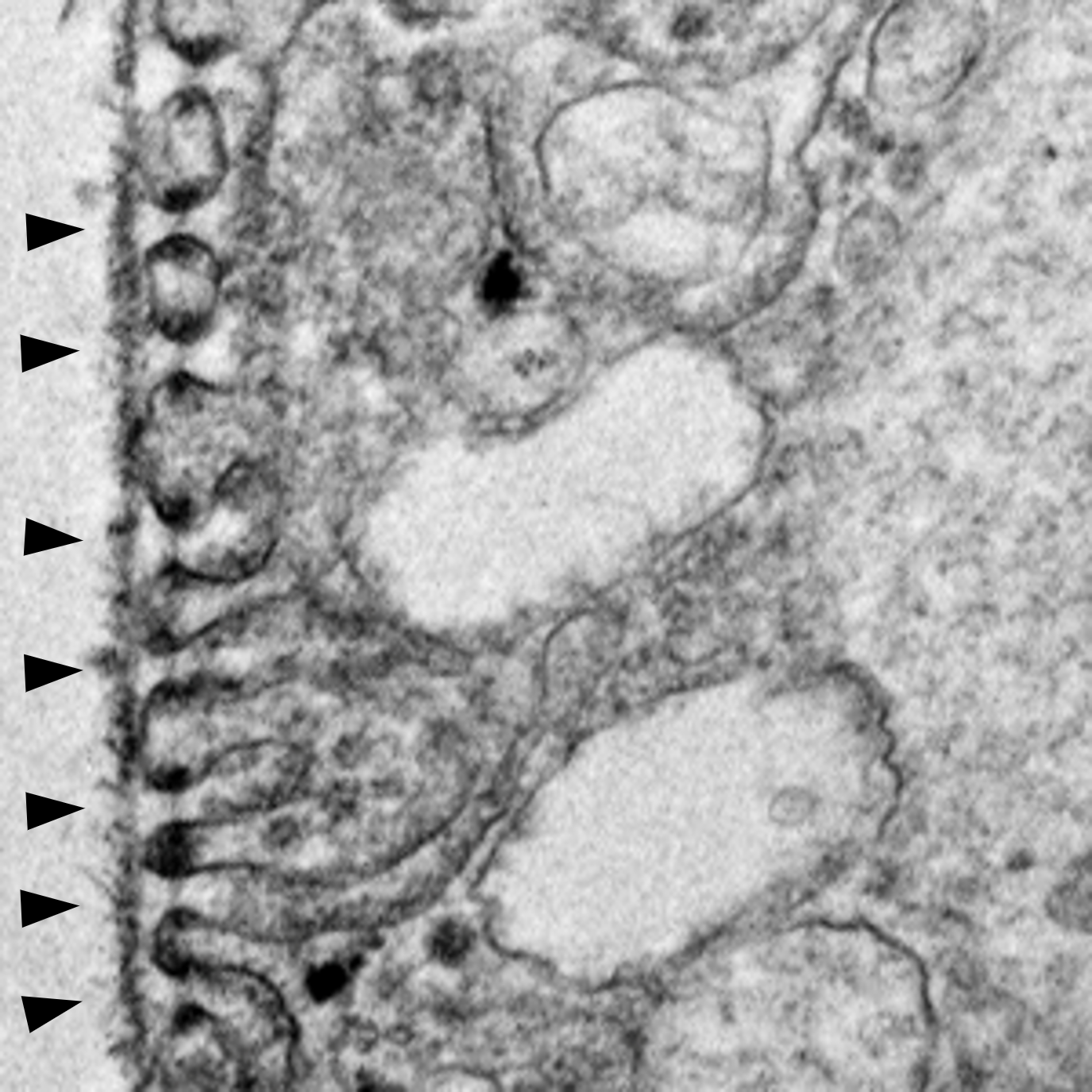

Supplement: Supplementary file 7 — Source data Fig. 4 [file 44318_2025_458_MOESM7_ESM.zip › EMBOJ-2024-119314R-SourceDataForFigure4/4G/Control.png]

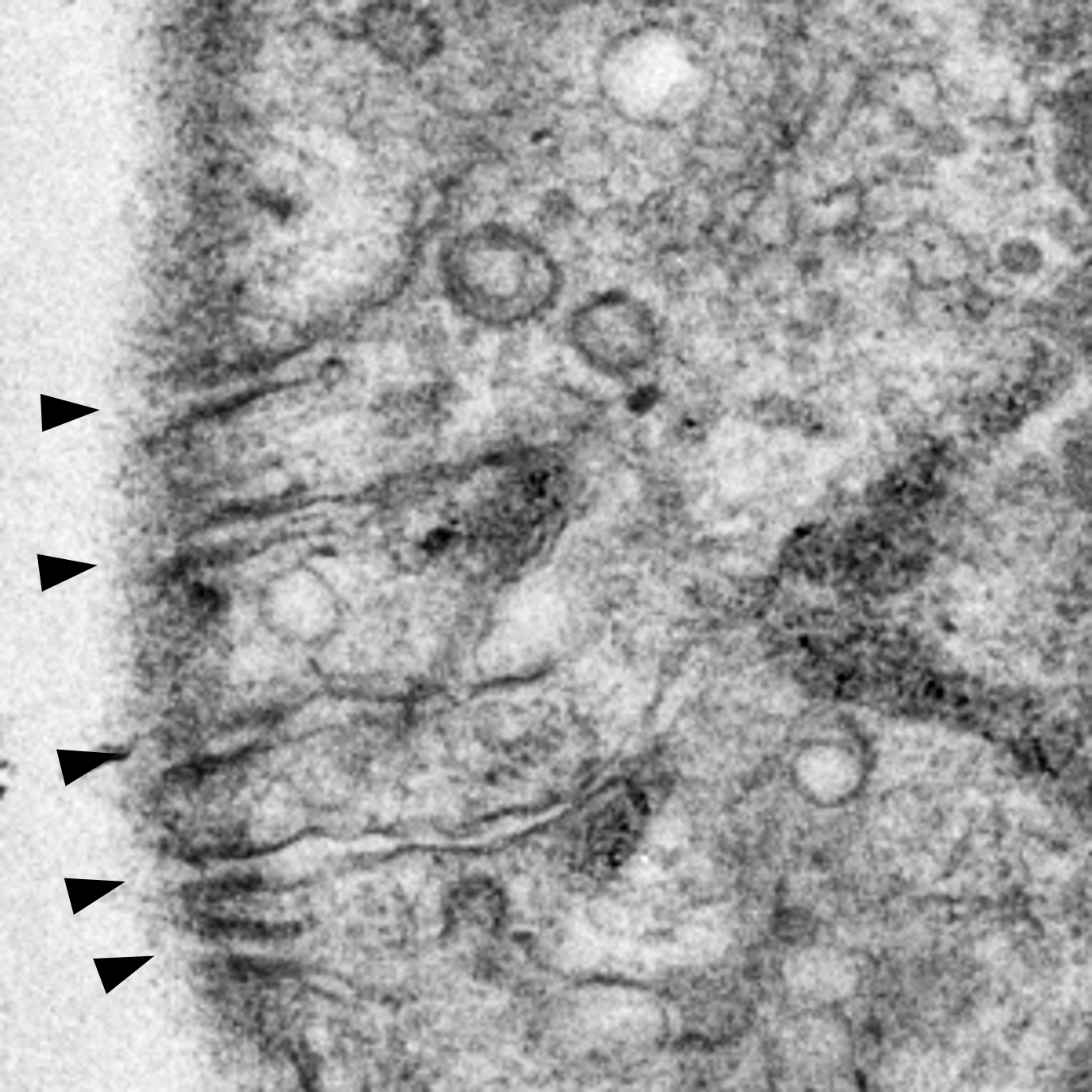

Supplement: Supplementary file 7 — Source data Fig. 4 [file 44318_2025_458_MOESM7_ESM.zip › EMBOJ-2024-119314R-SourceDataForFigure4/4G/Host.png]

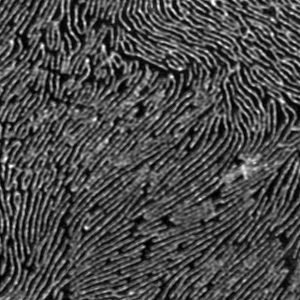

Supplement: Supplementary file 7 — Source data Fig. 4 [file 44318_2025_458_MOESM7_ESM.zip › EMBOJ-2024-119314R-SourceDataForFigure4/4H/Control.tif]

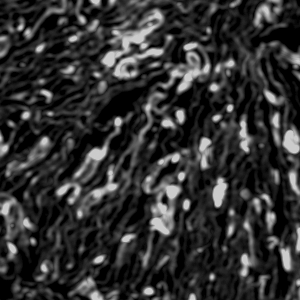

Supplement: Supplementary file 7 — Source data Fig. 4 [file 44318_2025_458_MOESM7_ESM.zip › EMBOJ-2024-119314R-SourceDataForFigure4/4H/Host.tif]

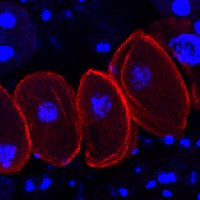

Supplement: Supplementary file 7 — Source data Fig. 4 [file 44318_2025_458_MOESM7_ESM.zip › EMBOJ-2024-119314R-SourceDataForFigure4/4J/Control-1.tif]

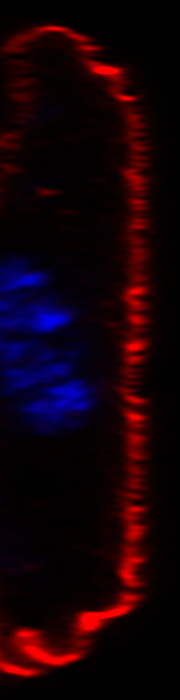

Supplement: Supplementary file 7 — Source data Fig. 4 [file 44318_2025_458_MOESM7_ESM.zip › EMBOJ-2024-119314R-SourceDataForFigure4/4J/Control-2.tif]

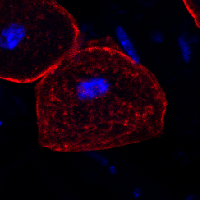

Supplement: Supplementary file 7 — Source data Fig. 4 [file 44318_2025_458_MOESM7_ESM.zip › EMBOJ-2024-119314R-SourceDataForFigure4/4J/Host-1.tif]

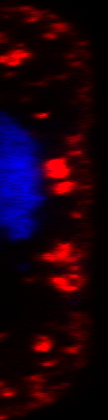

Supplement: Supplementary file 7 — Source data Fig. 4 [file 44318_2025_458_MOESM7_ESM.zip › EMBOJ-2024-119314R-SourceDataForFigure4/4J/Host-2.tif]

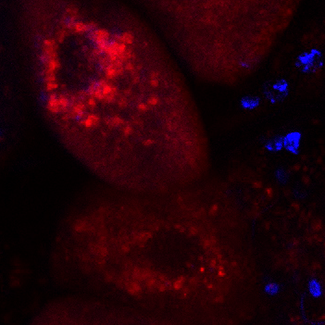

Supplement: Supplementary file 7 — Source data Fig. 4 [file 44318_2025_458_MOESM7_ESM.zip › EMBOJ-2024-119314R-SourceDataForFigure4/4K/Control.tif]

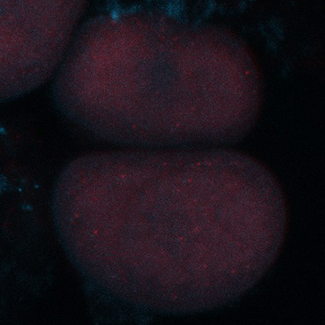

Supplement: Supplementary file 7 — Source data Fig. 4 [file 44318_2025_458_MOESM7_ESM.zip › EMBOJ-2024-119314R-SourceDataForFigure4/4K/Host.tif]

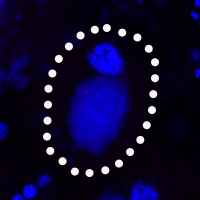

Supplement: Supplementary file 7 — Source data Fig. 4 [file 44318_2025_458_MOESM7_ESM.zip › EMBOJ-2024-119314R-SourceDataForFigure4/4M/Control-a.tif]

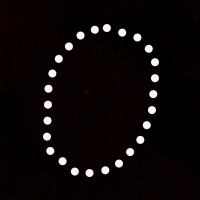

Supplement: Supplementary file 7 — Source data Fig. 4 [file 44318_2025_458_MOESM7_ESM.zip › EMBOJ-2024-119314R-SourceDataForFigure4/4M/Control-b.tif]

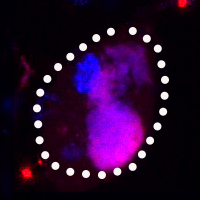

Supplement: Supplementary file 7 — Source data Fig. 4 [file 44318_2025_458_MOESM7_ESM.zip › EMBOJ-2024-119314R-SourceDataForFigure4/4M/Host-1-a.tif]

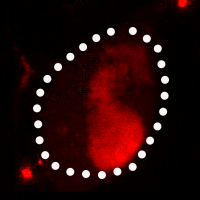

Supplement: Supplementary file 7 — Source data Fig. 4 [file 44318_2025_458_MOESM7_ESM.zip › EMBOJ-2024-119314R-SourceDataForFigure4/4M/Host-1-b.tif]

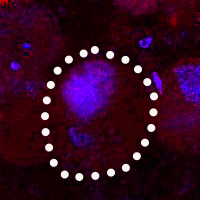

Supplement: Supplementary file 7 — Source data Fig. 4 [file 44318_2025_458_MOESM7_ESM.zip › EMBOJ-2024-119314R-SourceDataForFigure4/4M/Host-2-a.tif]

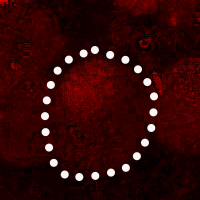

Supplement: Supplementary file 7 — Source data Fig. 4 [file 44318_2025_458_MOESM7_ESM.zip › EMBOJ-2024-119314R-SourceDataForFigure4/4M/Host-2-b.tif]

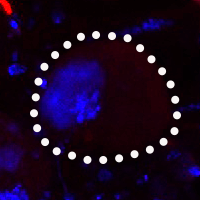

Supplement: Supplementary file 7 — Source data Fig. 4 [file 44318_2025_458_MOESM7_ESM.zip › EMBOJ-2024-119314R-SourceDataForFigure4/4M/Host-3-a.tif]

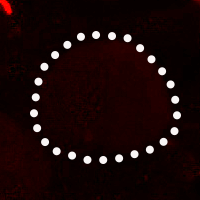

Supplement: Supplementary file 7 — Source data Fig. 4 [file 44318_2025_458_MOESM7_ESM.zip › EMBOJ-2024-119314R-SourceDataForFigure4/4M/Host-3-b.tif]

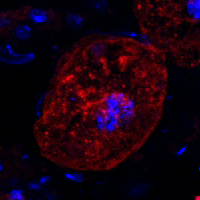

Supplement: Supplementary file 7 — Source data Fig. 4 [file 44318_2025_458_MOESM7_ESM.zip › EMBOJ-2024-119314R-SourceDataForFigure4/4O/Host.tif]

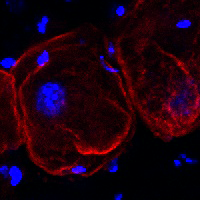

Supplement: Supplementary file 7 — Source data Fig. 4 [file 44318_2025_458_MOESM7_ESM.zip › EMBOJ-2024-119314R-SourceDataForFigure4/4O/sns-Rab5-IR Host.tif]

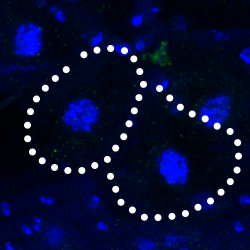

Supplement: Supplementary file 8 — Source data Fig. 5 [file 44318_2025_458_MOESM8_ESM.zip › EMBOJ-2024-119314R-SourceDataForFigure5/5A/Control-a.tif]

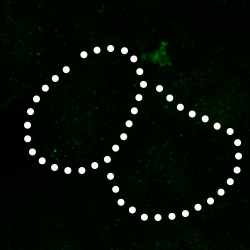

Supplement: Supplementary file 8 — Source data Fig. 5 [file 44318_2025_458_MOESM8_ESM.zip › EMBOJ-2024-119314R-SourceDataForFigure5/5A/Control-b.tif]

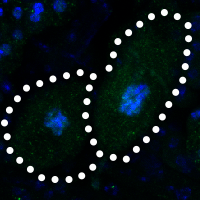

Supplement: Supplementary file 8 — Source data Fig. 5 [file 44318_2025_458_MOESM8_ESM.zip › EMBOJ-2024-119314R-SourceDataForFigure5/5A/Host-a.tif]

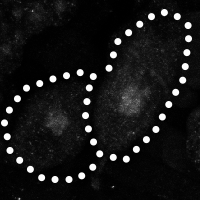

Supplement: Supplementary file 8 — Source data Fig. 5 [file 44318_2025_458_MOESM8_ESM.zip › EMBOJ-2024-119314R-SourceDataForFigure5/5A/Host-b.tif]

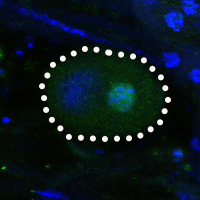

Supplement: Supplementary file 8 — Source data Fig. 5 [file 44318_2025_458_MOESM8_ESM.zip › EMBOJ-2024-119314R-SourceDataForFigure5/5B/Ap-a.tif]

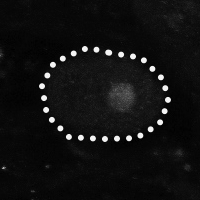

Supplement: Supplementary file 8 — Source data Fig. 5 [file 44318_2025_458_MOESM8_ESM.zip › EMBOJ-2024-119314R-SourceDataForFigure5/5B/Ap-b.tif]

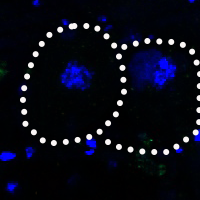

Supplement: Supplementary file 8 — Source data Fig. 5 [file 44318_2025_458_MOESM8_ESM.zip › EMBOJ-2024-119314R-SourceDataForFigure5/5B/PBS-a.tif]

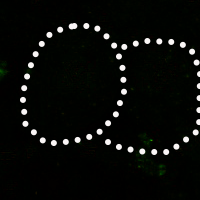

Supplement: Supplementary file 8 — Source data Fig. 5 [file 44318_2025_458_MOESM8_ESM.zip › EMBOJ-2024-119314R-SourceDataForFigure5/5B/PBS-b.tif]

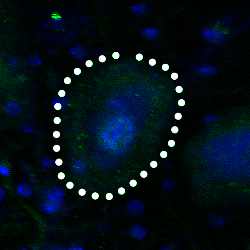

Supplement: Supplementary file 8 — Source data Fig. 5 [file 44318_2025_458_MOESM8_ESM.zip › EMBOJ-2024-119314R-SourceDataForFigure5/5B/PGN-a.tif]

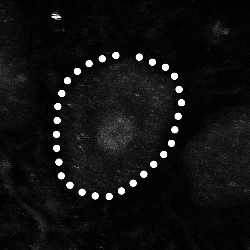

Supplement: Supplementary file 8 — Source data Fig. 5 [file 44318_2025_458_MOESM8_ESM.zip › EMBOJ-2024-119314R-SourceDataForFigure5/5B/PGN-b.tif]

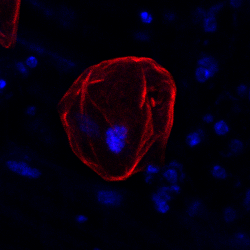

Supplement: Supplementary file 8 — Source data Fig. 5 [file 44318_2025_458_MOESM8_ESM.zip › EMBOJ-2024-119314R-SourceDataForFigure5/5C/PBS.tif]

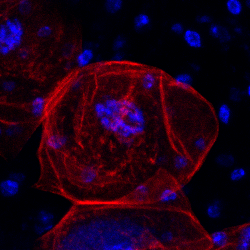

Supplement: Supplementary file 8 — Source data Fig. 5 [file 44318_2025_458_MOESM8_ESM.zip › EMBOJ-2024-119314R-SourceDataForFigure5/5C/PGN.tif]

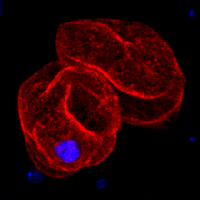

Supplement: Supplementary file 8 — Source data Fig. 5 [file 44318_2025_458_MOESM8_ESM.zip › EMBOJ-2024-119314R-SourceDataForFigure5/5E/Ap.tif]

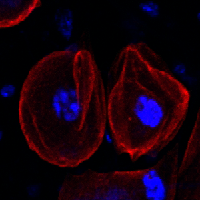

Supplement: Supplementary file 8 — Source data Fig. 5 [file 44318_2025_458_MOESM8_ESM.zip › EMBOJ-2024-119314R-SourceDataForFigure5/5E/PBS.tif]

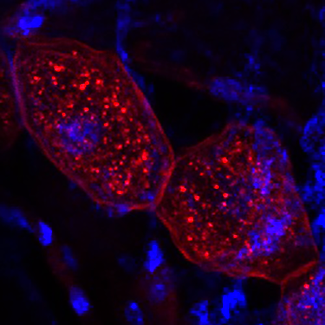

Supplement: Supplementary file 8 — Source data Fig. 5 [file 44318_2025_458_MOESM8_ESM.zip › EMBOJ-2024-119314R-SourceDataForFigure5/5G/Host.tif]

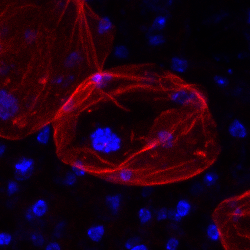

Supplement: Supplementary file 8 — Source data Fig. 5 [file 44318_2025_458_MOESM8_ESM.zip › EMBOJ-2024-119314R-SourceDataForFigure5/5G/sns-PGRP-LC-IR Host.tif]

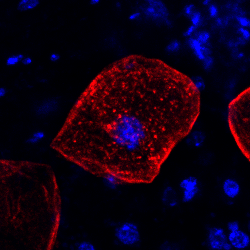

Supplement: Supplementary file 8 — Source data Fig. 5 [file 44318_2025_458_MOESM8_ESM.zip › EMBOJ-2024-119314R-SourceDataForFigure5/5J/Host.tif]

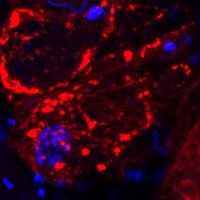

Supplement: Supplementary file 8 — Source data Fig. 5 [file 44318_2025_458_MOESM8_ESM.zip › EMBOJ-2024-119314R-SourceDataForFigure5/5J/sns-uas-PGRP-lc host.tif]

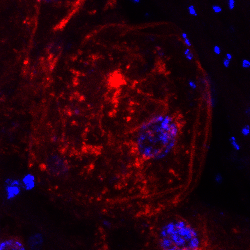

Supplement: Supplementary file 8 — Source data Fig. 5 [file 44318_2025_458_MOESM8_ESM.zip › EMBOJ-2024-119314R-SourceDataForFigure5/5J/sns-uas-PGRP-LC.tif]

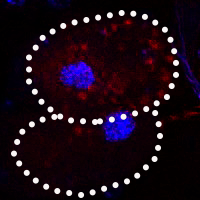

Supplement: Supplementary file 8 — Source data Fig. 5 [file 44318_2025_458_MOESM8_ESM.zip › EMBOJ-2024-119314R-SourceDataForFigure5/5L/sns-Gale.tif]

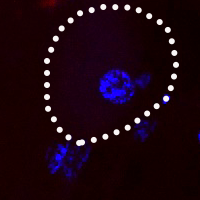

Supplement: Supplementary file 8 — Source data Fig. 5 [file 44318_2025_458_MOESM8_ESM.zip › EMBOJ-2024-119314R-SourceDataForFigure5/5L/sns-UAS-PGRP-LC.tif]

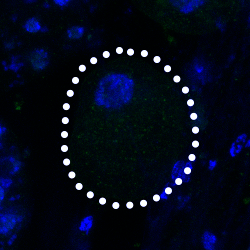

Supplement: Supplementary file 9 — Source data Fig. 6 [file 44318_2025_458_MOESM9_ESM.zip › EMBOJ-2024-119314R-SourceDataForFigure6/6A/ABF Host-a.tif]

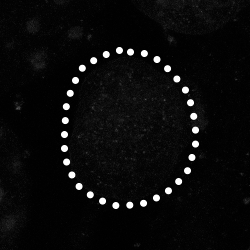

Supplement: Supplementary file 9 — Source data Fig. 6 [file 44318_2025_458_MOESM9_ESM.zip › EMBOJ-2024-119314R-SourceDataForFigure6/6A/ABF Host-b.tif]

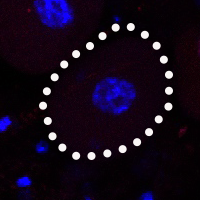

Supplement: Supplementary file 9 — Source data Fig. 6 [file 44318_2025_458_MOESM9_ESM.zip › EMBOJ-2024-119314R-SourceDataForFigure6/6B/ABF Host.tif]

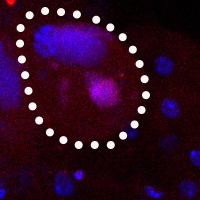

Supplement: Supplementary file 9 — Source data Fig. 6 [file 44318_2025_458_MOESM9_ESM.zip › EMBOJ-2024-119314R-SourceDataForFigure6/6B/Host.tif]

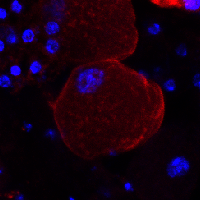

Supplement: Supplementary file 9 — Source data Fig. 6 [file 44318_2025_458_MOESM9_ESM.zip › EMBOJ-2024-119314R-SourceDataForFigure6/6C/ABF Host.tif]

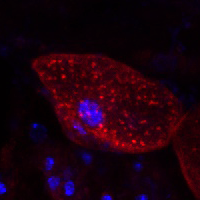

Supplement: Supplementary file 9 — Source data Fig. 6 [file 44318_2025_458_MOESM9_ESM.zip › EMBOJ-2024-119314R-SourceDataForFigure6/6C/Host.tif]

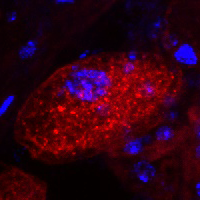

Supplement: Supplementary file 9 — Source data Fig. 6 [file 44318_2025_458_MOESM9_ESM.zip › EMBOJ-2024-119314R-SourceDataForFigure6/6F/A Host.tif]

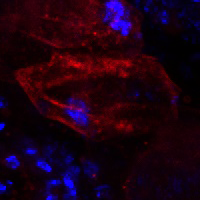

Supplement: Supplementary file 9 — Source data Fig. 6 [file 44318_2025_458_MOESM9_ESM.zip › EMBOJ-2024-119314R-SourceDataForFigure6/6F/GF Host.tif]

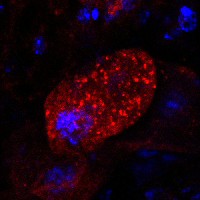

Supplement: Supplementary file 9 — Source data Fig. 6 [file 44318_2025_458_MOESM9_ESM.zip › EMBOJ-2024-119314R-SourceDataForFigure6/6F/Host.tif]

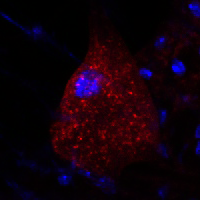

Supplement: Supplementary file 9 — Source data Fig. 6 [file 44318_2025_458_MOESM9_ESM.zip › EMBOJ-2024-119314R-SourceDataForFigure6/6F/L Host.tif]

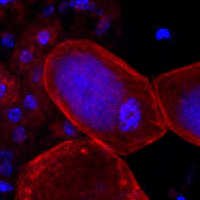

Supplement: Supplementary file 9 — Source data Fig. 6 [file 44318_2025_458_MOESM9_ESM.zip › EMBOJ-2024-119314R-SourceDataForFigure6/6H/AST-120 Host.png]

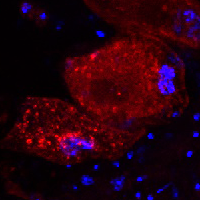

Supplement: Supplementary file 9 — Source data Fig. 6 [file 44318_2025_458_MOESM9_ESM.zip › EMBOJ-2024-119314R-SourceDataForFigure6/6H/Host.tif]
