# Supplementary material for: Translocation of gut bacteria promotes tumor-associated mortality by inducing immune-activated renal damage
Source: EMBO J. 2025 May 22;44(13):3586–613. doi: 10.1038/s44318-025-00458-5 (PMC12217037; doi:10.1038/s44318-025-00458-5)
Supplement: Supplementary file 11 — Expanded View Figures [file 44318_2025_458_MOESM11_ESM.pdf]

Expanded View Figures

Figure EV1. Low- and high- passage NICD-TZ tumors have distinct effects on the host.

(**A**) Schematic diagram of the shearing of wild-type salivary-gland imaginal rings (IMR) and injection into the control host abdomen. (**B**) Comparison of the lifespan of control host flies injected with either wild-type IMR or SD medium. SD, $n = 58$; WT-IMR, $n = 57$. (**C, D**) The abdominal cavity of host flies carrying GFP-labeled G1 (**C**) or G43 (**D**) tumors. Scale bar, 200 µm. (**E**) Comparison of tumor sizes with different tumor passages. Box plots are defined as follows: Box plots are defined as follows: Primary Tumor—min: 1126, lower whisker: 1126, 25th percentile: 2426, median: 3130, mean: 3334, 75th percentile: 3808, upper whisker: 5881, max: 7606. No-inject—min: 0, lower whisker: 0, 25th percentile: 0, median: 0, mean: 0, 75th percentile: 0, upper whisker: 0, max: 0. G1 Tumor—min: 6047, lower whisker: 6047, 25th percentile: 37,428, median: 48,503, mean: 56,475, 75th percentile: 70,092, upper whisker: 119,087, max: 150,350. Cut Tumor—min: 841, lower whisker: 841, 25th percentile: 2844, median: 3691, mean: 3901, 75th percentile: 4640, upper whisker: 7335, max: 8026. G5 Tumor—min: 42,124, lower whisker: 42,124, 25th percentile: 135,722, median: 181,204, mean: 195,632, 75th percentile: 272,787, upper whisker: 345,174, max: 345,174. G6 Tumor—min: 1010, lower whisker: 1010, 25th percentile: 36,995, median: 72979, mean: 72,979, 75th percentile: 108,963, upper whisker: 144,947, max: 144,947. G7 Tumor—min: 24862, lower whisker: 24,862, 25th percentile: 41,071, median: 183483, mean: 165,166, 75th percentile: 234,836, upper whisker: 372,677, max: 372,677. G8 Tumor—min: 60,425, lower whisker: 60,425, 25th percentile: 87,598, median: 198,436, mean: 221,770, 75th percentile: 328,039, upper whisker: 501,919, max: 501,919. G9 Tumor—min: 33,675, lower whisker: 33,675, 25th percentile: 74,424, median: 122,889, mean: 127,351, 75th percentile: 177,309, upper whisker: 257,638, max: 257,638. G10 Tumor—min: 2505, lower whisker: 2505, 25th percentile: 70,678, median: 174,480, mean: 175,830, 75th percentile: 259,220, upper whisker: 484,617, max: 484,617.G0, $n = 32$; Non-inject, $n = 23$; G1, $n = 32$; Cut, $n = 36$; G5, $n = 12$; G6, $n = 2$; G7, $n = 15$; G8, $n = 10$; G9, $n = 9$; G10, $n = 22$. (**F**) Comparison of the lifespan of tumor host flies with increasing tumor passages. G1, $n = 43$; G10, $n = 55$; G15, $n = 49$; G20, $n = 55$; G29, $n = 56$. (**G**) Relative triglyceride levels in control and G1 host whole flies (tumor removed) normalized to protein amounts. Three groups were repeated. ns $p = 0.1574$. (**H**) Relative trehalose levels in control and G1 host whole flies (tumor removed) normalized to extracted protein amounts. Three groups were repeated. ns $p = 0.6143$. (**I**) Tumor hosts with varying abdomen sizes. Scale bar, 500 µm. Data is presented as mean ± SEM, Student's t test. Source data are available online for this figure.

© The Author(s)

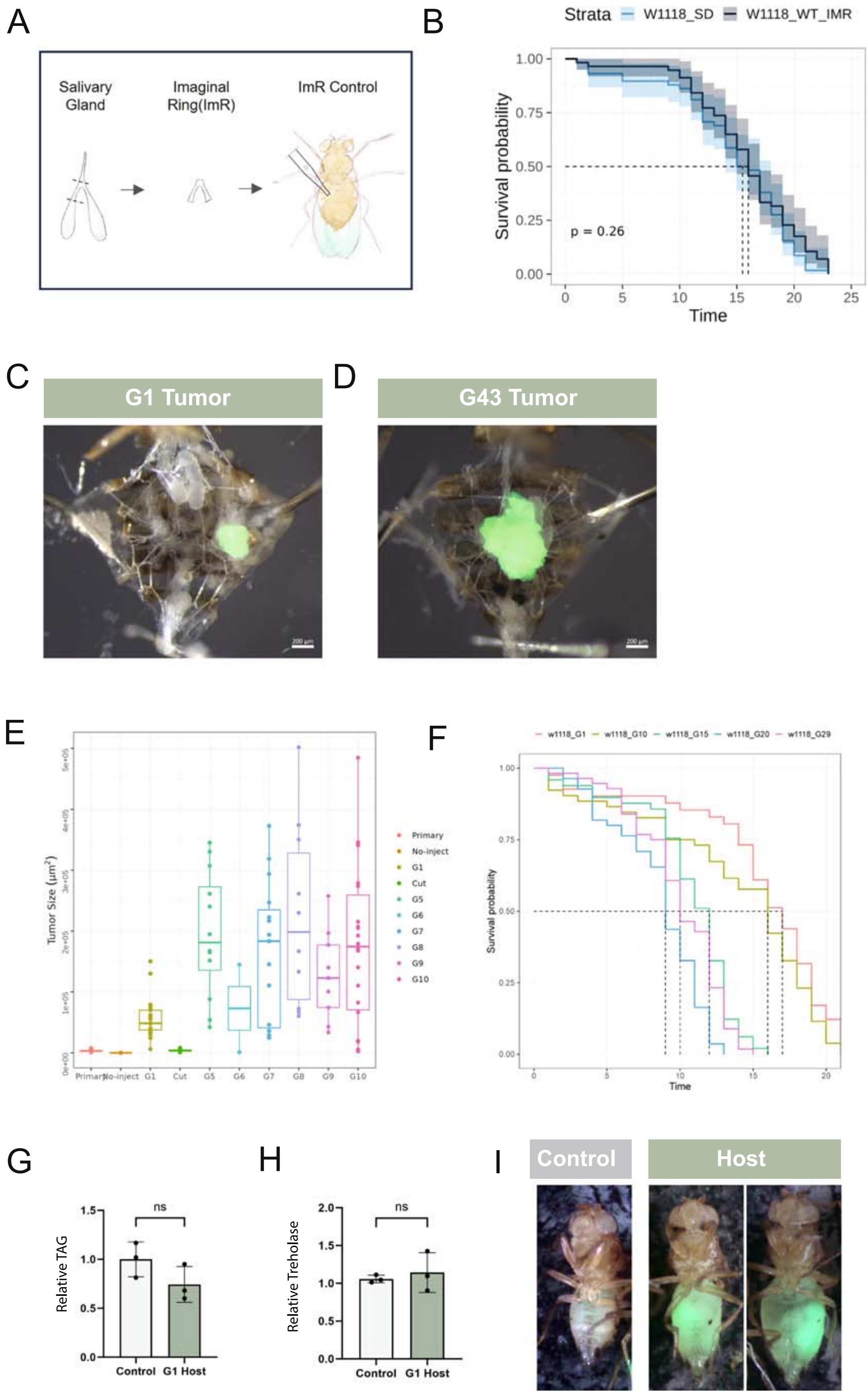
A
Salivary Gland
Imaginal Ring(ImR)
ImR Control
B
Strata W1118_SD W1118_WT_IMR
1.00
0.75
0.50
0.25
0.00
Survival probability
p = 0.26
0 5 10 15 20 25
Time
C
G1 Tumor
D
G43 Tumor
E
Tumor Size (μm²)
Primary No-inject G1 Cut G5 G6 G7 G8 G9 G10
Primary
No-inject
G1
Cut
G5
G6
G7
G8
G9
G10
F
w1118_G1 w1118_G10 w1118_G15 w1118_G20 w1118_G29
Survival probability
Time
G
Relative TAG
ns
Control G1 Host
H
Relative Treholase
ns
Control G1 Host
I
Control
Host

 © The Author(s)

A

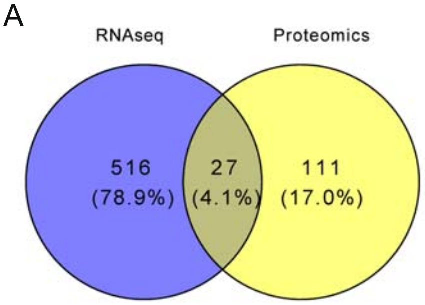

B

Gene Name	Gene Name	Gene Name
CG4269	CG3328	Ance-3
CG10814	AttC	Mtk
Spn88Eb	frma	DptB
PGRP-SA	fat-spondin	Nepl15
Idgf1	AttD	Diedel
Irc	Nepl13	CG34423
CG32032	Cht4	fon
CG1208	AttA	CG13026
CG3604	hgo	phu

C

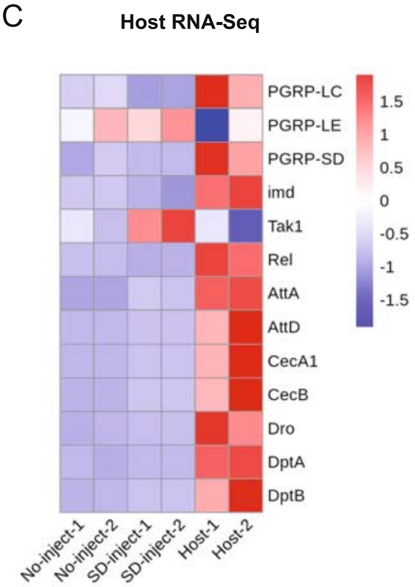

Figure EV2. Both RNA-seq and proteomic analyses show elevated immune response in tumor hosts.

(**A**) Venn diagram illustrates the overlap between upregulated genes identified through RNA-seq and proteomic analyses in tumor hosts. (**B**) List of shared upregulated genes from both RNA-seq and proteomic analyses. (**C**) Heatmap showing Imd pathway gene expression in the flies without injection (No-inject), control hosts injected with SD medium (SD-inject), and tumor hosts (Host).

© The Author(s)

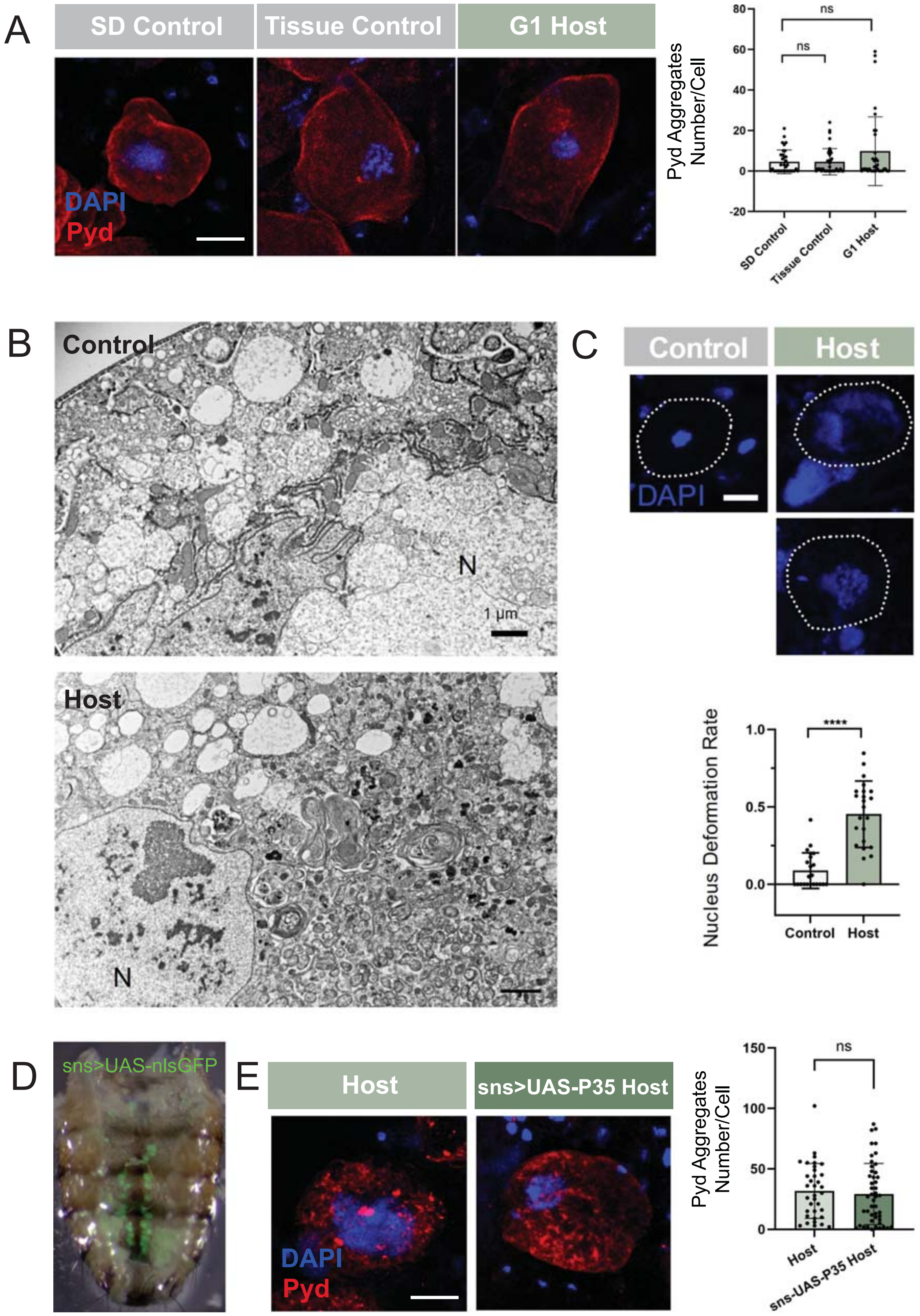
A
SD Control
Tissue Control
G1 Host
DAPI
Pyd
Pyd Aggregates Number/Cell
ns
ns
80
60
40
20
0
-20
SD Control
Tissue Control
G1 Host
B
Control
N
1 µm
Host
N
C
Control
Host
DAPI
Nucleus Deformation Rate
****
1.0
0.5
0.0
Control
Host
D
sns>UAS-nlsGFP
E
Host
sns>UAS-P35 Host
DAPI
Pyd
Pyd Aggregates Number/Cell
ns
150
100
50
0
Host
sns-UAS-P35 Host

 © The Author(s)

Figure EV3. : NICD-TZ tumors cause nephrocyte damage and death in the host.

(**A**) Pyd staining pattern and aggregate statistical data of SD-injected controls, Normal Tissue injected controls and G1 hosts. Scale bar, 20 µm. SD control, $n = 34$; Tissue control, $n = 37$; G1 host, $n = 34$. ns lower $p = 0.9634$. ns upper $p = 0.0918$. (**B**) TEM images of control and tumor host nephrocytes. "N" indicates nucleus. Scale bar, upper 1 µm, lower 2 µm. (**C**) Nuclear morphology in control and tumor-host nephrocytes. The white dots outline the nephrocyte shape. Scale bar, 15 µm. Quantification of defective nuclear morphology ratio in control ($n = 22$) and tumor-host flies ($n = 23$). ****$p = 1.11E\text{-}08$. (**D**) Expression of *sns-GAL4 > nlsGFP* in pericardial nephrocytes in adult flies. (**E**) Pyd staining pattern and aggregates statistical data of hosts and *sns-Gal4 > UAS-P35* hosts. Scale bar, 20 µm. Host, $n = 35$; sns>P35, $n = 47$. ns $p = 0.6423$. Data is presented as mean ± SEM, Student's t test. Source data are available online for this figure.

© The Author(s)

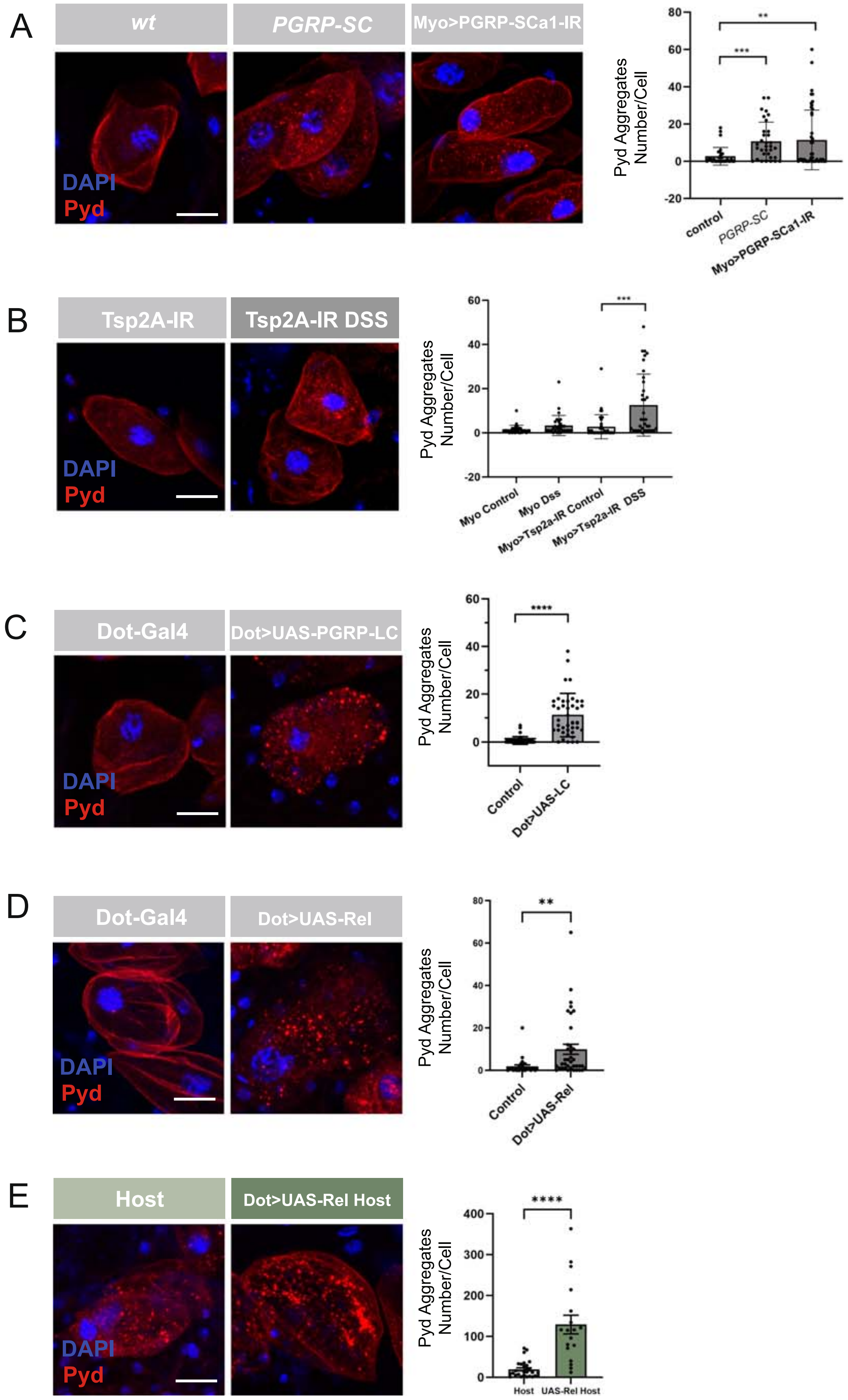
A
wt
PGRP-SC
Myo>PGRP-SCa1-IR
DAPI
Pyd
Pyd Aggregates Number/Cell
control
PGRP-SC
Myo>PGRP-SCa1-IR
B
Tsp2A-IR
Tsp2A-IR DSS
Myo Control
Myo Dss
Myo>Tsp2a-IR Control
Myo>Tsp2a-IR DSS
C
Dot-Gal4
Dot>UAS-PGRP-LC
Control
Dot>UAS-LC
D
Dot>UAS-Rel
Dot>UAS-Rel
E
Host
Dot>UAS-Rel Host
Host
UAS-Rel Host

Figure EV4. The nephrocyte immune response leads to mis-localization of Pyd on the cell membrane.

(**A**) Pyd staining in wild-type flies, *PGRP-SC*$^{\Delta}$ mutations and *Myo1A-Gal4; Gal80∧ts > PGRP-SCa1-RNAi* nephrocytes. Quantification of Pyd aggregates is shown on the right. *wt*, $n = 32$; *PGRP-SC*$^{\Delta}$, $n = 33$; *Myo1A-Gal4; Gal80∧ts > PGRP-SCa1-RNAi*, $n = 45$. Scale bar, 20 µm. $^{**}p = 0.0036$, $^{***}p = 0.0001$. (**B**) Pyd staining in *Myo1A-Gal4; Gal80∧ts> Tsp2A-RNAi* fly in normal food and in DSS added food. Quantification of Pyd aggregates is shown on the right. Myo control, $n = 30$; Myo DSS, $n = 30$; *Tsp2A-RNAi*, $n = 38$; *Tsp2A-RNAi* DSS, $n = 35$. Scale bar, 20 µm. $^{***}p = 0.0002$. (**C**) Pyd staining in *Dot-GAL4* and *Dot-GAL4 > UAS-PGRP-LC* nephrocytes. Quantification of Pyd aggregates is shown on the right. Dot-GAL4, $n = 40$; *Dot-GAL4 > UAS-PGRP-LC*, $n = 40$. Scale bar, 20 µm. $^{****}p = 2.25E\text{-}10$. (**D**) Pyd staining in *Dot-GAL4* and *Dot-GAL4 > UAS-Relish* nephrocytes. Quantification of Pyd aggregates is shown on the right. Dot-GAL4, $n = 29$; *Dot-GAL4 > UAS-Relish*, $n = 37$. Scale bar, 20 µm. $^{**}p = 0.0044$. (**E**) Pyd staining in *Dot-Gal4* Host and *Dot-GAL4 > UAS-Relish* tumor host nephrocytes. Quantification of Pyd aggregates is shown on the right. Dot-GAL4 host, $n = 28$; *Dot-GAL4 > UAS-Relish* Host, $n = 18$. Scale bar, 20 µm. $^{****}p = 5.96E\text{-}10$. Data is presented as mean ± SEM, Student's t test. Source data are available online for this figure.

© The Author(s)

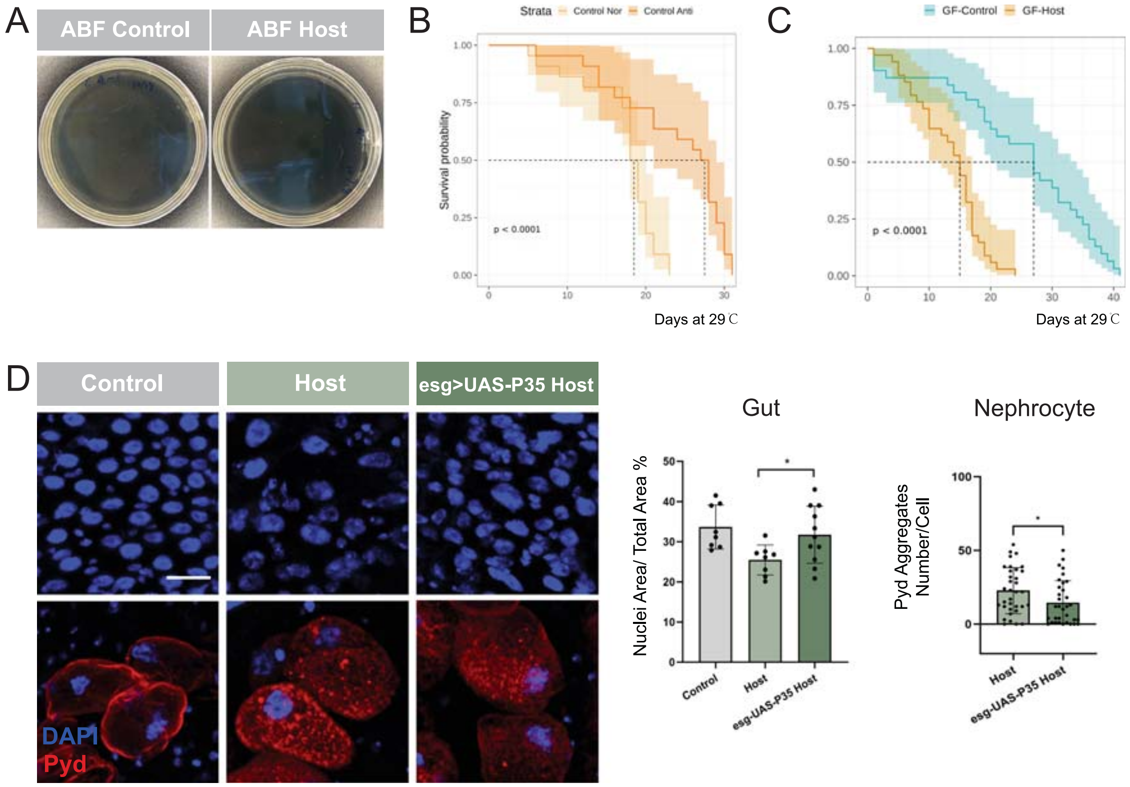

Figure EV5. Reducing bacterial load and improving gut health benefit nephrocyte function and extend host survival time.

(**A**) Intestinal bacterial load of antibiotic treated control and tumor host flies. (**B**) Lifespan of controls fed with normal food and antibiotic food. Control, $n = 22$; Control Anti, $n = 22$. ****$p = 4E-05$. (**C**) Lifespan of control and tumor host flies raised in germ-free conditions (GF). GF-Control, $n = 28$; GF-Host, $n = 34$. ****$p = 2E-15$. (**D**) DAPI staining of midgut in control, Host and *esg-Gal4; Gal80∧ts > UAS-P35* host flies. Quantification of nuclei densities is shown on the left. Control, $n = 8$; Host, $n = 8$; *esg > UAS-P35* Host, $n = 11$. Pyd staining in control, Host and *esg-Gal4; Gal80∧ts > UAS-P35* host nephrocytes. Quantification of Pyd aggregates is shown on the right. Host, $n = 34$; *esg > UAS-P35* Host, $n = 33$. Scale bar, 20 µm. Gut *$p = 0.0361$, Nephrocyte *$p = 0.0336$. Data is presented as mean ± SEM, Student's t test. Source data are available online for this figure.

 © The Author(s)
